# Essential roles of mitochondrial biogenesis regulator Nrf1 in retinal development and homeostasis

**DOI:** 10.1186/s13024-018-0287-z

**Published:** 2018-10-17

**Authors:** Takae Kiyama, Ching-Kang Chen, Steven W Wang, Ping Pan, Zhenlin Ju, Jing Wang, Shinako Takada, William H Klein, Chai-An Mao

**Affiliations:** 1 Ruiz Department of Ophthalmology and Visual Science, McGovern Medical School at The University of Texas Health Science Center at Houston (UTHealth), 6431 Fannin St., MSB 7.024, Houston, TX 77030 USA; 2 Department of Ophthalmology, Baylor College of Medicine, 1 Baylor Plaza, Houston, TX 77030 USA; 3 Department of Systems Biology, The University of Texas MD Anderson Cancer Center, 1515 Holcombe Blvd, Houston, TX 77030 USA; 4 Department of Bioinformatics and Computational Biology, The University of Texas MD Anderson Cancer Center, 1515 Holcombe Blvd, Houston, TX 77030 USA; 5 Department of Biochemistry and Molecular Biology, The University of Texas MD Anderson Cancer Center, 1515 Holcombe Blvd, Houston, TX 77030 USA; 6 Present Address: Office of Scientific Review, National Institute of General Medical Sciences, National Institutes of Health, Bethesda, MD 20892 USA

**Keywords:** Mitochondrial biogenesis, Nrf1, Retinal progenitor cell, Retinal ganglion cell, Optic atrophy, Photoreceptor degeneration

## Abstract

**Background:**

Mitochondrial dysfunction has been implicated in the pathologies of a number of retinal degenerative diseases in both the outer and inner retina. In the outer retina, photoreceptors are particularly vulnerable to mutations affecting mitochondrial function due to their high energy demand and sensitivity to oxidative stress. However, it is unclear how defective mitochondrial biogenesis affects neural development and contributes to neural degeneration. In this report, we investigated the in vivo function of nuclear respiratory factor 1 (Nrf1), a major transcriptional regulator of mitochondrial biogenesis in both proliferating retinal progenitor cells (RPCs) and postmitotic rod photoreceptor cells (PRs).

**Methods:**

We used mouse genetic techniques to generate RPC-specific and rod PR-specific *Nrf1* conditional knockout mouse models. We then applied a comprehensive set of tools, including histopathological and molecular analyses, RNA-seq, and electroretinography on these mouse lines to study Nrf1-regulated genes and Nrf1’s roles in both developing retinas and differentiated rod PRs. For all comparisons between genotypes, a two-tailed two-sample student’s *t*-test was used. Results were considered significant when *P* < 0.05.

**Results:**

We uncovered essential roles of Nrf1 in cell proliferation in RPCs, cell migration and survival of newly specified retinal ganglion cells (RGCs), neurite outgrowth in retinal explants, reconfiguration of metabolic pathways in RPCs, and mitochondrial morphology, position, and function in rod PRs.

**Conclusions:**

Our findings provide in vivo evidence that Nrf1 and Nrf1-mediated pathways have context-dependent and cell-state-specific functions during neural development, and disruption of Nrf1-mediated mitochondrial biogenesis in rod PRs results in impaired mitochondria and a slow, progressive degeneration of rod PRs. These results offer new insights into the roles of Nrf1 in retinal development and neuronal homeostasis and the differential sensitivities of diverse neuronal tissues and cell types of dysfunctional mitochondria. Moreover, the conditional *Nrf1* allele we have generated provides the opportunity to develop novel mouse models to understand how defective mitochondrial biogenesis contributes to the pathologies and disease progression of several neurodegenerative diseases, including glaucoma, age-related macular degeneration, Parkinson’s diseases, and Huntington’s disease.

## Background

Mitochondrial biogenesis is a dynamic subcellular process through which existing mitochondria continuously import and integrate new proteins and lipids, replicate mitochondrial DNA (mtDNA), and fuse and divide upon environment changes. This process is intricately regulated to maintain a healthy mitochondrial network, essential for energy homeostasis, metabolism, signaling, and apoptosis. The vast majority of the ~ 1500 proteins involved in mitochondrial structure and function are encoded by nuclear genes, which are regulated in concert with a set of transcriptional regulators, including peroxisome proliferative activated receptor gamma coactivator 1 (PGC-1) family members, nuclear respiratory factor 1 (Nrf1), and nuclear respiratory factor 2 (Nrf2/GABP) [1–4].

*Nrf1* encodes an evolutionarily conserved transcription activator [5–9]. Nrf1 binds to GC-rich DNA elements in promoters of many nuclear genes required for mitochondrial biogenesis and respiratory function [9–11]. In primary cortical neurons, Nrf1 has been shown to co-regulate all cytochrome c oxidase (COX) subunits and several glutamatergic neurochemicals, implying that a Nrf1-mediated higher-order mechanism coordinately controls the expression of genes involved in neuronal activity and energy metabolism [12–15]. In muscle, Nrf1 has been shown to be a direct PGC-1 target, the master regulator of mitochondrial biogenesis, whose dysfunction has been implicated in several neurodegenerative diseases, such as Parkinson’s disease [1, 4]. In addition, Nrf1 plays a significant role in cell growth and proliferation. A recent study using chromatin immunoprecipitation sequencing (ChIP-seq) analysis identified 2470 potential Nrf1 targets in human neuroblastoma cells, indicating roles for Nrf1 in regulating genes for mitochondrial biogenesis and cell growth and in the pathogenesis of neurodegenerative diseases [16]. Interestingly, several genes involved in the glycolytic pathway, such as PFKB2, PGAM1, PGKM5, and ALDOA, were also found in this list, suggesting a possible Nrf1 role in reprogramming metabolic processes. Nrf1 also interacts with several proteins involved in different cellular functions. For example, it interacts directly with poly(ADP-ribose) polymerase 1 (PARP-1), and PARP-1 modulates Nrf1’s DNA-binding domain for transcriptional regulation [17]. Dynein light chain was also shown to interact with NRF-1, although the functional significance remains unknown [18].

Several in vivo studies have revealed distinct functions of *Nrf1* in different developing organisms. In zebrafish, an insertional mutation in the *Nrf1* locus caused a cell death phenotype in developing photoreceptors [7]. In Drosophila, the *Nrf1* homolog gene *erect wing* (*ewg*) has been shown to regulate Hippo pathway activity in a neuronal subtype-specific manner to determine neuronal fate in developing retinas [19]. In mice, *Nrf1*-null embryos fail to maintain mtDNA and die between embryonic day 3.5 (E3.5) and 6.5 [20]. These studies offer insights into the understanding of Nrf1’s in vivo function in different developmental systems and cellular context, but how Nrf1-regulated pathways function in retinal development and how they contribute to defective mitochondrial biogenesis to affect neural development and contribute to neural degeneration is unknown.

In this report, we studied the function of Nrf1 during mouse retinal development. We show that *Nrf1* is expressed in proliferating retinal progenitor cells (RPCs) in embryonic retinas and enriched in retinal ganglion cells (RGCs) and rod photoreceptors cells (PRs), both of which consume large amounts of energy. Using cell-type-specific *Nrf1* knockout mice, we demonstrate that *Nrf1* controls cell proliferation in RPCs and the extension of neurite processes in developing retinal neurons. Nrf1-deficient embryonic retinas exhibited affected expression of genes involved in multiple cellular processes. In differentiated rod PRs, deleting *Nrf1* caused abnormal mitochondrial morphology, deteriorated mitochondrial functions, abnormal photoreceptor inner and outer segments, and reduced electroretinography (ERG) activities. Eventually, mutant rod PRs completely degenerated. Together, these results demonstrate the crucial role of Nrf1-mediated mitochondrial biogenesis in retinal development and homeostasis and provide new insights into Nrf1 function in neurite outgrowth and metabolic reprogramming.

## Methods

### Gene targeting and animal breeding

**Fig. 1:**
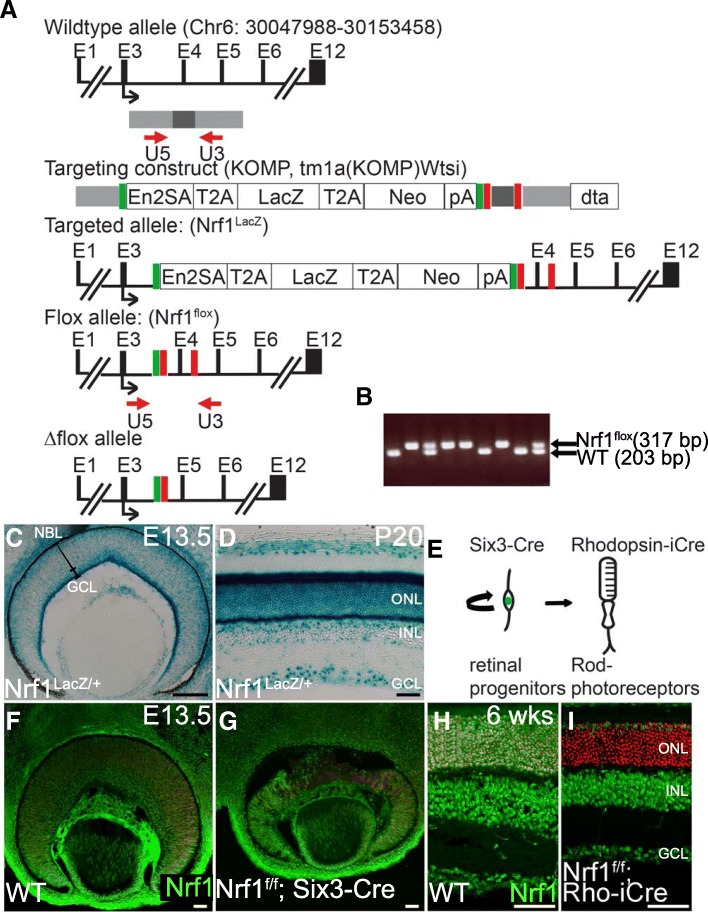
Generation of Nrf1 expression and conditional *Nfr1* alleles. (**a**) Genomic structure of *Nrf1*, the targeting construct, the targeted *Nrf1*^*LacZ*^ and *Nrf1*^*flox*^ alleles, and the deleted allele. Exons are indicated as E1-E12. The gray and black bars indicate the regions used in the targeting construct. A black arrow indicates the translational start site for the Nrf1 protein. Red arrows indicate PCR primers used for PCR genotyping of the wildtype and floxed alleles. Red boxes indicate loxP sites, and green boxes indicate FRT sites. (**b**) PCR genotyping using U5 and U3 primers for wildtype (203 bp) and floxed (317 bp) alleles. (**c**, **d**) Nrf1 expression during retinogenesis revealed by LacZ expression in *Nrf1*^*LacZ/+*^ retinas at E13.5 (**c**) and P20 (**d**). (**e**) Schematic representation of the developing retinal cells expressing Six3 and rhodopsin. Proliferative retinal progenitor cells expressing Six3 will give rise to all mature retinal cells. Rhodopsin is expressed in differentiated rod photoreceptor cells. (**f–i**) Nrf1 expression detected by immunofluorescent staining on E13.5 wildtype (**f**) and *Nrf1*^*f/f*^*;Six3-Cre* (**g**) retinal sections and on 6-week-old wildtype (**h**) and *Nrf1*^*f/f*^*;Rho-iCre* (**i**) retinal sections. Scale bars: 100 μm in C, 50 μm in **d**-**i**. ONL: outer nuclear layer. INL: inner nuclear layer. GCL: ganglion cell layer. WT: wildtype. NBL: neural blast layer

A Nrf1-targeted embryonic stem (ES) clone was obtained from the knockout mouse project repository (http://www.mousephenotype.org/data/alleles/MGI:1332235/tm1a(KOMP)Wtsi). This allele contains 2 loxP sites inserted into the third and fourth introns, and a FLP recombinase target (FRT)-site flanked T2A-LacZ-T2A-neomycin fusion cassette inserted into intron 3. Exon 4 in the floxed allele can be deleted by Cre-mediated recombination. ES cells were injected into B6(GC)-Tyrc-2 J/J blastocysts, and the injected blastocysts were transferred into C57/BL6 albino females. Chimeric males obtained by blastocyst injection were bred to wildtype B6(GC)-Tyrc-2 J/J females to generate the *Nrf1*^*LacZ/+*^ allele, which was subsequently bred with a 17T17T*Rosa26-FLPeR*0T17T0T17T 0T0Ttransgene to remove a *LacZ-neomycin* fusion cassette to generate a *Nrf1*^*flox/+*^ allele (Fig. 1a). PCR primers used to distinguish the *Nrf1*^*flox*^ allele from the wildtype allele were U5 (5’-CCAAGACTTGTATGCATTGGTCTCAG-3′) and U3 (5’-GCACTTCTGGCTCCATGGTCC-3′) (Fig. 1a, b). PCR primers for *Six3-Cre* were Cre1 (5′-AACGAGTGATGAGGTTCGCAAGAAC-3′) and Cre2 (5′-CGCTATTTTCCATGAGTGAACGAACC-3′); and for *Rho-iCre* were iCre1 (5’-GGATGCCACCTCTGATGAAG-3′) and iCre2 (5’-CACACCATTCTTTCTGACCCG-3′). Embryos were designated as E0.5 at noon on the day in which vaginal plugs were observed. Both male and female mice were used in this study, and no differences were observed according to sex.

### Histology, immunohistochemistry, X-gal staining, COX activity

Embryos or eyeballs dissected from mice were fixed in 4% paraformaldehyde at 4 °C for 2 h or overnight, embedded in paraffin or optimal cutting temperature (OCT) compound, and sectioned into 7 μm thickness for histological analysis. After dewaxing and rehydration, the sections were stained with Hematoxylin and Eosin.

For immunohistochemical analysis, cryo- or paraffin-embedded embryos or eyes were sectioned into 7 μm or 30 μm thickness. Sections were heat-treated in a microwave oven at 600 W in 10 mM sodium citrate for 15 min. The sections were blocked with 2% bovine serum albumin and 5% normal serum for 2 h at room temperature. The primary antibody was applied to the sections for 1–3 days at 4^o^C. The primary antibodies used were mouse anti-Nrf1 (1:300, catalog #PCRP-NFR1-3D4; DSHB, The University of Iowa, Iowa City, IA), mouse anti-Isl1 (1:200, catalog# 37.3F7; DSHB), goat anti-Brn3/Pou4f2 (1:150, catalog #sc-6026; Santa Cruz Biotechnology, Dallas, TX), mouse anti-Pax6 (1:200, catalog #MAB5552; Chemicon, Burlington, MA), sheep anti-Chx10 (1:300, catalog #X1180P; Exalpha, Shirley, MA), rabbit anti-cleaved caspase-3 (1:300, catalog #9579; Cell Signaling, Danvers, MA), mouse anti-BrdU (1:10, catalog #05–633; Millipore, Burlington, MA), mouse anti-Phospho-Histone H3/PH3 (1:700, catalog #9706; Cell Signaling), rabbit anti-Cyclin D1 (1:300, catalog #MA1–39546; Thermo Fisher Scientific, Waltham, MA), mouse anti-rhodopsin (1:20, catalog #MS-1233-R7; Thermo Fisher Scientific), rabbit anti-cone arrestin (1:2000, catalog #AB16282; Millipore), and rabbit anti-Tfam (1:500, catalog #ab131607; Abcam, Cambridge, MA). Secondary antibodies conjugated with Alexa-488, 555 or 633 (Thermo Fisher Scientific) were used in 1:800 dilution. For indirect immunofluorescence, a tyramide signal amplification kit was used (PerkinElmer, Waltham, MA). HRP-conjugated secondary antibodies were from Jackson ImmunoResearch Laboratories (West Grove, PA). DAPI (2.5 μg/ml, catalog #D1306; Thermo Fisher Scientific) was used to stain nuclei. Images were captured using Olympus (Tokyo, Japan) FluoView 1000 or Zeiss (Thornwood, NY) LSM 780 confocal microscopes. SimplePCI software (Hamamatsu Corporation, Sewickley, PA) was used to analyze the number of cells.

For X-gal staining, embryos or eyes were fixed in 10% formalin for 30 min, embedded in OCT compound, and sectioned into 30 μm thickness. Sections were dried at room temperature for 3 h, washed with wash buffer (0.1 M sodium phosphate containing 2 mM MgCl_2_, 0.01% deoxycholate, and 0.02% Nonidet P-40). LacZ color reaction was performed in wash buffer containing 5 mM potassium ferrocyanide, 5 mM potassium ferricyanide, and 1 mg/ml X-gal at 37^o^C overnight. Color reaction was terminated by incubation in 10% formalin for 10 min. Post-fixed sections were washed, dehydrated, and mounted with Cytoseal 60 (Thermo Fisher Scientific). Images were collected with a Canon EOS 10 digital camera (Melville, NY) mounted on an Olympus IX71 microscope.

Cytochrome c oxidase (COX) analysis was performed as described previously [21] with slight modifications. E13.5 embryonic heads from wildtype and *Nrf1*^*f/f*^*;Six3-Cre* embryos or 6 week-old adult eyeballs from wildtype and *Nrf1*^*f/f*^*;Rho-iCre* were fixed in 10% formalin for 20 min at room temperature. Samples were washed in phosphate buffered saline (PBS) 3 times and embedded in OCT; 14 μm cryo-sections were collected. Sections were dried at 4^o^C for 1 hour, rehydrated, and incubated in COX reacting solution (1× DAB, 100 μM cytochrome C, and 2 μg/ml bovine catalase in 0.1 M PBS, pH 7.0) at 37^o^C. Color reactions were terminated by incubating in 10% formalin for 10 min. Post-fixed sections were washed, dehydrated, and mounted with Cytoseal 60. Images were collected as described for X-gal staining.

### BrdU labeling and TUNEL assays

Terminal deoxynucleotidyl transferase dUTP nick end label (TUNEL) assays were performed using an in situ cell death detection kit (Roche, Pleasanton, CA). For pulse labeling with BrdU, 0.1 mg per body gram of BrdU (Sigma, St. Louis, MO) was injected intraperitoneally into pregnant females 1 h before embryo collection.

### RNA sequencing analysis

Eighteen retinas from wildtype and *Nrf1*^*f/f*^*;Six3-Cre* embryos at E13.5 of multiple littermates were pooled, and RNA was extracted using TRI reagent (Sigma) and purified with a Pure Link RNA mini kit (Thermo Fisher Scientific). RNA sequencing (RNA-seq) was performed in the Sequencing and Microarray Core Resource Facility at The University of Texas MD Anderson Cancer Center. RNAs were treated with DNase, and cDNAs were synthesized using a cDNA synthesis kit (NuGen, San Carlos, CA). One hundred nt paired-end reads were obtained using an Illumina HiSeq 3000 Next Generation Sequencing instrument (San Diego, CA). The RNA-seq experiment was duplicated, thus making statistical comparisons possible, although there is still a lack of statistical power. The RNA-seq reads were mapped to the mouse genome (mm10) via the Tophat 2.7.2 program. We performed QC and sum counts (reads) for each gene using HTseq. The differential expression analyses were performed by Cuffdiff software. Nrf1-dependent genes (fold change ≥ 1.4; adjusted *P*-value ≤ 0.2) were analyzed with DAVID Bioinformatics Resources 6.8 (https://david.ncifcrf.gov/home.jsp). The raw datasets and normalized count data for each gene have been deposited in NCBI (GSE101550).

### Retinal explant culture

Retinal explant culture was described previously [22]. In brief, retinas were isolated from E13.5 wildtype and *Nrf1*^*f/f*^*;Six3-Cre* embryos and cut in 4 pieces, then placed on laminin-coated coverslips and cultured in Neurobasal media containing N2 supplement and penicillin-streptomycin (Thermo Fisher Science). Images were examined and collected using an Olympus IX-70 inverted microscope.

### In situ hybridization

Embryo heads from wildtype and *Nrf1*^*f/f*^*;Six3-Cre* at E13.5 were dissected and fixed in fresh 10% neutral buffered formalin for 24 h. Samples were washed with PBS then dehydrated with serial ethanol and embedded in paraffin. Sections were cut to 7 μm or 10 μm in thickness. In situ hybridization was performed as described previously [23]. Antisense *Idh1* (957 bp) and *Ldha* (950 bp) probes were cloned by reverse transcriptase PCR using Idh1 probe F (5’-AGGTTCTGTGGTGGAGATGC-3′), Idh1 probe R (5’-GACGTCTCTTGCCCTTTCTG-3′), Ldha probe F (5’-TCCGTTACCTGATGGGAGAG-3′) and Ldha probe R (5’-ACACTTGGGTGGTTGGTTCC-3′). RNAscope in situ hybridization (ISH) was performed using the RNAscope 2.5 HD Detection Reagents-Brown kit following manufacturer’s protocol (cat# 322310, Advanced Cell Diagnostics, Newark, CA). According to instructions, each mRNA molecule hybridized to a probe appears as a separate brown color dot. The probes used were mouse Cpt1a-C1 (cat# 443071) and mouse Slc16a1-C1 (cat# 423661).

### Quantitative reverse transcriptase PCR (qRT-PCR)

Eight retinas from wildtype or *Nrf1*^*f/f*^*;Six3-Cre* embryos at E13.5 or one retina from wildtype or *Nrf1*^*f/f*^*; Rho-iCre* at 6 weeks old of multiple littermates were pooled, and RNAs were extracted using TRI reagent (Sigma). First-strand cDNA was synthesized using the Superscript III First-Strand Synthesis System (Thermo Fisher Scientific). Real-time PCR was performed using CFX Connect Real-Time System (BioRad, Hercules, CA) with SsoAdvanced Universal SYBR Green Supermix (BioRad). Relative RNA levels were normalized to that of *β-actin*. Sequences of PCR primers are listed in Table 1.

**Table 1:** Primer sets used for qRT-PCR

Gene	Position	5′-sequence-3’
b-actin	Forward	CAACGGCTCCGGCATGTGC
	Reverse	CTCTTGCTCTGGGCCTCG
CCND1	Forward	GCACTTTTGGTCAGCTAGCT
	Reverse	GACATGGCCCTAAACCTTCT
Gli1	Forward	ACTGGGGTGAGTTCCCTTCT
	Reverse	AGGACTACCCAGCAAATCCT
Mapt	Forward	AATGGAAGACCATGCTGGAG
	Reverse	TCCCAATCTGAGTCCCAAAG
Ret	Forward	TGGCACACCTCTGCTCTATG
	Reverse	CTGTTCCCAGGAACTGTGGT
Stmn3	Forward	CCCGAACACCATCTACCAGT
	Reverse	CTTCTGCAGCTCTTCCAAGG
Ncan	Forward	GTGGCTGCTTCTCCTAGTGG
	Reverse	AATGTCTCGCAGGGAGCTTA
Ina	Forward	TTCGGGAATACCAGGACTTG
	Reverse	GTGCTAAACCGCGTCTCTTC
Islr2	Forward	CTCTGCCTTTTCAAGGATGC
	Reverse	CGCTGAGTTGAAAGGCCTAC
Nell2	Forward	CACAGTTGACCTTTCCTGCT
	Reverse	CAGCACAAATGGCCATTCTT
Stmn2	Forward	GCAATGGCCTACAAGGAAAA
	Reverse	GGTGGCTTCAAGATCAGCTC
Gap43	Forward	GTGCTGCTAAAGCTACCACT
	Reverse	CTTCAGAGTGGAGCTGAGAA
Nrn1	Forward	CCAGGGGAATGACTTCAAGA
	Reverse	TTTCGCTTTTCTGGAGGAGA
Syt4	Forward	TGTTGTAGGTGATGGTTTCA
	Reverse	AGACCATGGTTCTTAGGTGA
Dcx	Forward	ACAGATGTCAACCGGGAAAG
	Reverse	TCGTTCGTCAAAATGTCCAA
Pou4f1	Forward	AGGCCTATTTGCCGTACAA
	Reverse	CGTCTCACACCCTCCTCAGT
Irx4	Forward	GAGACCACCAGCACACTGAA
	Reverse	AGGTGGAAACCTGTGTGAGG
Cx3cr1	Forward	AGCCCAGGGGAAGAAATAGA
	Reverse	CTCTGTTGGCTCCAGTCTCC
Capn3	Forward	GCTTCTGGAGGAAGACGATG
	Reverse	TTTGGGAACCTCGTAGATGG
Igfbp7	Forward	GGAAAATCTGGCCATTCAGA
	Reverse	TGCGTGGCACTCATACTCTC
Vit	Forward	GCGTCTACGCGTCTTACTCC
	Reverse	CCCTTTTGGGGCTTACTTTC
Nid1	Forward	ACCATCACCTTCCAGGAGTG
	Reverse	GCATAGCGCAAGATCCTCTC
Stab1	Forward	ACAAGATCTTCAGCCGCCTA
	Reverse	AGTTTGTCACGGTGGTCCTC
Spp1	Forward	TGCACCCAGATCCTATAGCC
	Reverse	CTCCATCGTCATCATCATCG
Tgfbi	Forward	GGATGTCCTGAAGGGAGACA
	Reverse	ATTGGTGGGAGCAAAAACAG
Cox4i2	Forward	AGCTGAGCCAAGCAGAGAAG
	Reverse	GCCCATCACTGTCTTCCATT
Idh1	Forward	AGGTTCTGTGGTGGAGATGC
	Reverse	GACGCCCACGTTGTATTTCT
Dna2	Forward	CGAAGTTCTGTGCATCCTGA
	Reverse	TTCTCAGACACCGAATGCTG
Gdap1	Forward	CTGTGAGGCCACTCAGATCA
	Reverse	TGAGCTCAGGATGCAAAATG
Shmt2	Forward	CTCTTTGCTTCGGACCACTC
	Reverse	TTCTCCCTCTGCAGAAGCTC
Cpt1a	Forward	CCAGGCTACAGTGGGACATT
	Reverse	AAGGAATGCAGGTCCACATC
Sardh	Forward	ACTCGGTTGTCTTCCCACAC
	Reverse	CCTGTCGCTCTTGAAACACA
Ucp2	Forward	GCCACTTCACTTCTGCCTTC
	Reverse	GAAGGCATGAACCCCTTGTA
Pmaip1	Forward	CCCAGATTGGGGACCTTAGT
	Reverse	AGTTATGTCCGGTGCACTCC
Rab32	Forward	CTCTTCTCCCAGCACTACCG
	Reverse	CAAATGCTCCAAGAGCTTCC
Ldha	Forward	AGGCTCCCCAGAACAAGATT
	Reverse	TCTCGCCCTTGAGTTTGTCT
HK1	Forward	GAAGCCAAATGGGACTGTGT
	Reverse	CACGCACAGATTGGTTATGC
Pfkp	Forward	GAAGCCAAATGGGACTGTGT
	Reverse	CACGCACAGATTGGTTATGC
Tpi1	Forward	CCTGGCCTATGAACCTGTGT
	Reverse	CAGGTTGCTCCAGTCACAGA
Pgam2	Forward	AGGAGCTGCCTACCTGTGAA
	Reverse	GGGCTGCAATAAGCACTCTC
Mfn1	Forward	GCTGTCAGAGCCCATCTTTC
	Reverse	CAGCCCACTGTTTTCCAAAT
Mfn2	Forward	GTCCTGGACGTCAAAGGGTA
	Reverse	GCAGAACTTTGTCCCAGA
Opa1	Forward	GATGACACGCTCTCCAGTGA
	Reverse	TCGGGGCTAACAGTACAACC

### Transmission electron microscopy

Eyeballs were fixed with 3% glutaraldehyde and 2% paraformaldehyde overnight at 4 °C. Retinas were washed and treated with 0.1% cacodylate-buffered tannic acid, post-fixed with 1% osmium tetroxide, stained en bloc with 1% uranyl acetate, and dehydrated with an ethanol gradient series. The samples were embedded in epon and sectioned with a JLB ultracut microtome (Leica, Wetzlar, Germany). Images were examined with a JEM 1010 REM (JEOL, Peabody, MA) and collected digitally. Fiji was used to analyze the size and circularity of inner segment (IS) and mitochondria [24].

### Mitochondrial DNA quantitation

Quantification of the relative copy number of mitochondrial DNA present per nuclear genome was performed as previously described [25]. Mitochondrial DNA and genomic Pecam DNA were amplified and analyzed by quantitative PCR (ΔΔC(t) method). PCR primers used to amplify mitochondrial DNA were mtDNAf (5’-CCTATCACCCTTGCCATCAT-3′) and mtDNAr (5’-GAGGCTGTTGCTTGTGTGAC-3′). PCR primers to amplify nuclear DNA were Pecamf (5’-ATGGAAAGCCTGCCATCATG-3′) and Pecamr (5’-TCCTTGTTGTTCAGCATCAC-3′).

### Electroretinography (ERG)

Mice were dark-adapted overnight and then anesthetized under infrared illumination by ketamine/xylazine/acepromazine through intraperitoneal injection (94/5/1 mg/kg), and pupils were dilated with 1% tropicamide and 2.5% phenylephrine topical eye drops (Bausch & Lomb, Tampa, FL). Body temperature was maintained at 35 °C to 37 °C by circulating 43.5 °C water through a plastic heating coil wrapped around the body. Stimulus-dependent transcorneal potential changes from both eyes were recorded simultaneously (UTAS BigShot system; LKC Technologies, Gaithersburg, MD) following the delivery of a white light flash with an intensity of 25 Candela sec m^−2^, as described previously [26]. The interstimulus interval was 120 s, and responses from 3 independent measurements were averaged and analyzed. Photopic ERG recordings ensued immediately after scotopic recordings by exposing the animals to a white background light of 30 Candela m^−2^ for 10 min. Transcorneal potential changes were then elicited by flashes of 25 Candela sec m^−2^ in intensity and presented at 1 Hz for 90 s, averaged, and then analyzed. A typical recording session lasted 1 h, and 5 μl of sterile-filtered PBS was applied every 20 min to ensure good electrical contact and delay the formation of corneal clouding and cataract.

### Experimental design

Six E13.5 littermate embryos of each genotype (wildtype and *Nrf1*^*f/f*^*;Six3-Cre*) were used for counting BrdU+ and PH3+ cells’ RPCs. Four sections near the central retinas from each embryo were stained with BrdU or PH3. The most representative sections from each embryo were used for cell counting. Total RNAs were isolated and pooled from 9 pairs of E13.5 wildtype and *Nrf1*^*f/f*^*;Six3-Cre* retinas for RNA-seq. For RNA-seq data validation using qRT-PCR, 3 sets of 6 pairs of E13.5 wildtype and *Nrf1*^*f/f*^*;Six3-Cre* retinas were isolated and pooled, and 2 independent experiments were conducted. For each qRT-PCR experiment, total RNAs from 4 pairs of E13.5 wildtype and *Nrf1*^*f/f*^*;Six3-Cre* retinas were isolated and pooled, and 5 sets of independent experiments were conducted. The ratio of *Nrf1*^*f/f*^*;Six3-Cre* to wildtype expression was calculated for each experiment and averaged for further analysis. For counting photoreceptors, 20 littermates from each genotype (wildtype and *Nrf1*^*f/f*^*;Rho-iCre*) were used for the study. Five littermates per genotype were sacrificed, and 4 sections from the central area of each retina were stained. One representative section from each sample was used to count the number of rows of photoreceptors at each study time point (3, 6, 7, and 8 weeks). For electron microscopy analysis, 2 pairs of wildtype and *Nrf1*^*f/f*^*;Rho-iCre* littermates were used. Images were collected from both mouse retinas. Four images of the IS of each genotype were used to quantify the shape and size of the IS and number of mitochondria. For ERG, 3 pairs of wildtype and *Nrf1*^*f/f*^*;Rho-iCre* littermates were used.

### Statistical analysis

All data are presented as mean ± standard deviation for each genotype. For all comparisons between genotypes, a two-tailed two-sample student’s *t*-test was used for all measurements. Results were considered significant when *P* < 0.05. Statistical tests were conducted using Excel (Microsoft, Redmond, WA).

## Results

### Nrf1 expression in the developing retina

To determine the expression and function of Nrf1 in the retina, we generated *Nrf1*^*LacZ*^ and Nrf1^flox^ targeted mouse lines (Fig. 1a, b). The *Nrf1*^*LacZ*^ knock-in allele contains a *LacZ* cassette, which was used to trace the spatiotemporal expression of *Nrf1*. We first examined the expression of *Nrf1* in developing and adult retinas. In E13.5 developing retinas, strong LacZ activity was detected near the apical and basal layers of the neural retina, suggesting Nrf1 is highly expressed in developing RGCs and photoreceptor precursor cells (Fig. 1c). Notably, weaker LacZ activity could also be detected in the neuroblast layer where proliferating RPCs and postmitotic precursor cells reside (Fig. 1c). Along the developmental progression, a similar pattern of LacZ expression was observed in E16.5 and P0 retinas (data not shown). In adult retinas, robust LacZ activity was observed in the ganglion cell layer and outer nuclear layer (ONL), where the metabolic activity is high (Fig. 1d) [27], while a moderate level of LacZ activity was observed in the inner nuclear layer (INL). The dynamic expression pattern of Nrf1 in both developing and mature retinas suggests that Nrf1 may play multiple roles in proliferating RPCs in the developing retina and in the differentiated retinal neurons.

To determine the functions of Nrf1 in RPCs and differentiated retinal neurons, we performed conditional knockout of *Nrf1* by breeding the *Nrf1*^*flox*^ allele with either *Six3-Cre* to delete *Nrf1* in the proliferating RPCs or with *Rho-iCre* to delete *Nrf1* in the rod PRs, respectively (Fig. 1e). During retinal development, the Six3-Cre transgenic line begins to activate Cre expression in the central retina at E11 [28], and the *Rho-iCre* transgenic line starts to activate Cre activity in differentiated rod PRs at P7 [26]. By immunostaining, Nrf1 protein was detected in developing RGCs and photoreceptor precursor cells, as well as in the neuroblast layer at E13.5 (Fig. 1f) and in cells in all nuclear layers in adult retinas (Fig. 1h). Consistent with the onset of Cre expression in both lines, the expression of Nrf1 protein was completely abolished in the central *Nrf1*^*f/f*^*; Six3-Cre* retina (compare Fig. 1f and g) and in the rod PRs of *Nrf1*^*f/f*^*; Rho-iCre* retinas (compare Fig. 1h and g) respectively, suggesting effective conditional deletion of *Nrf1* in RPCs by *Six3-Cre* or in rod PRs by *Rho-iCre*.

### Deleting Nrf1 in RPCs causes RGC loss and retinal degeneration

**Fig. 2:**
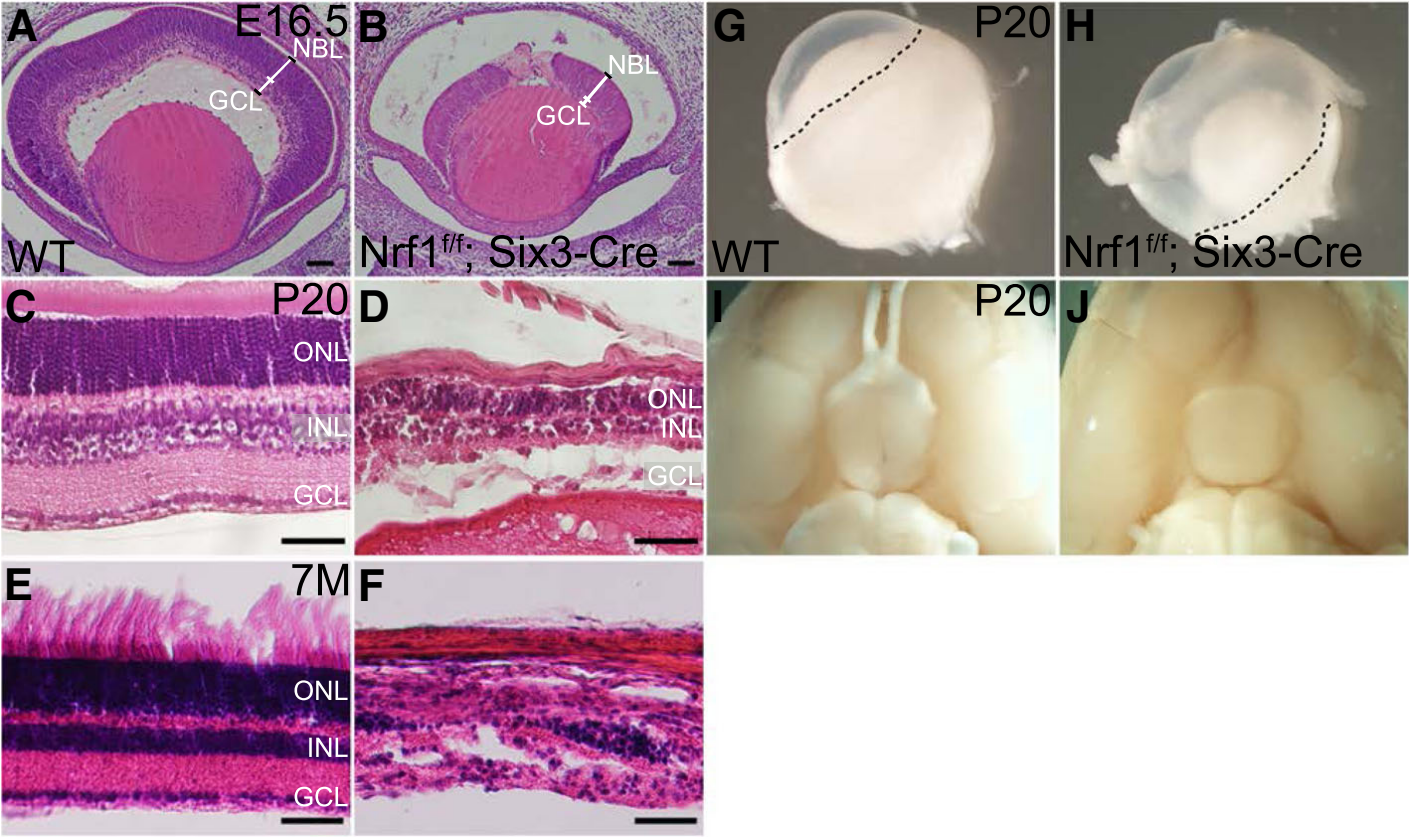
Loss of retinal ganglion cells and severe retinal degeneration in Nrf1-deficient RPCs. (**a–f**) Hematoxylin and eosin staining of retinal sections from wildtype (**a**, **c** and **e**) and *Nrf1*^*f/f*^*;Six3-Cre* (**b**, **d**, and **f**) at E16.5, P20, and 7 months old. (**g**, **h**) Eyeballs from wildtype (**g**) and *Nrf1*^*f/f*^*;Six3-Cre* (**h**) animals. The peripheral rim of underlying retinas is plotted with dotted lines. (**i**, **j**) Ventral view of the brains showing the optic nerve and optic chiasm in wildtype (**i**) and *Nrf1*^*f/f*^*;Six3-Cre* (**j**) animals. Scale bars: 50 μm. ONL: outer nuclear layer. INL: inner nuclear layer. GCL: ganglion cell layer. WT: wildtype. NBL: neural blast layer

To determine whether deleting *Nrf1* in embryonic retinas affects retinal development, we first examined the histology and morphology of Six3-Cre-mediated *Nrf1* mutant retinas (*Nrf1*^*f/f*^*;Six3-Cre*) at different developmental stages. At E16.5, *Nrf1*^*f/f*^*; Six3-Cre* retinas were substantially smaller and thinner than those of the wildtype retinas (Fig. 2a, b), causing a large sub-retinal space between the retina and the pigmented epithelium. The central regions of *Nrf1*^*f/f*^*;Six3-Cre* retinas near the optic disc were completely disrupted and acellular (arrowheads in Fig. 2b). At P20, *Nrf1*^*f/f*^*;Six3-Cre* retinas were relatively thinner than those of wildtype retinas. Decreased cell numbers in all cellular layers were observed with near complete abolishment of RGCs. Although the stereotypic laminar structure was retained in *Nrf1*-mutant retinas, the cells in each laminar layer were not properly aligned as in control retinas (Fig. 2c, d). In 7-month-old *Nrf1*^*f/f*^*;Six3-Cre* retinas, the number of retinal cells was further reduced, and the laminar layers were completely disrupted (Fig. 2e, f). The surface of the whole retina from *Nrf1*^*f/f*^*;Six3-Cre* was much smaller, underlying only a limited area near the optic disc in the eyeball (Fig. 2g, h). There were no visible optic nerves or optic chiasms in *Nrf1*^*f/f*^*;Six3-Cre* mice (Fig. 2i, j). These data suggest that deleting Nrf1 in RPCs causes substantial RGC loss followed by the degeneration of the entire retina.

### Delayed RGC differentiation, defective RGC migration, and apoptotic RGCs in *Nrf1*^*f/f*^*;Six3-Cre* retinas

Because a significant loss of RGCs was seen in *Nrf1*^*f/f*^*;Six3-Cre* retinas, we examined whether and how the RGC differentiation program was affected. RGCs are the first retinal cell type to differentiate from Atoh7-expressing precursor cells during retinogenesis. RGC differentiation is marked by the onset of Pou4f2 and Isl1 expression in the central retina around E12 [29, 30]. To determine when RGCs began to differentiate in *Nrf1*^*f/f*^*;Six3-Cre* retina, we monitored Isl1 and Pou4f2 expression by immunostaining at different embryonic stages.

**Fig. 3:**
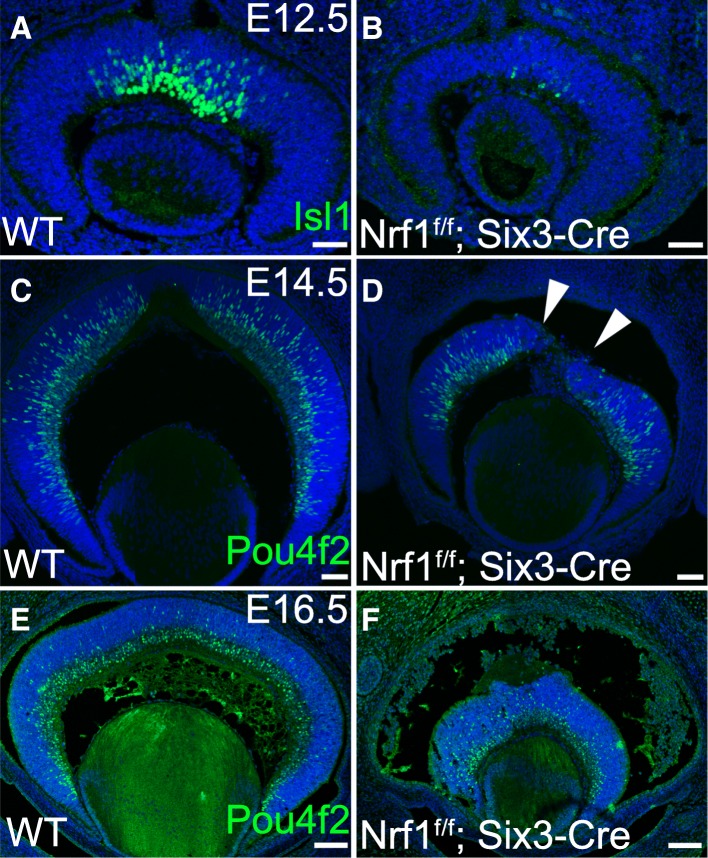
Delayed onset of RGC differentiation in *Nrf1*^*f/f*^*;Six3-Cre* retina. (**a–f**) Immunostaining of wildtype (**a**, **c**, and **e**) and *Nrf1*^*f/f*^*;Six3-Cre* (**b**, **d**, and **f**) retinas. (**a**, **b**) E12.5 retinal sections labeled with anti-Isl1 antibody. (**c**, **d**) E14.5 and (**e**, **f**) E16.5 retinal sections labeled with anti-Pou4f2 antibody. Arrowheads indicate clumped Pou4f2+ cells in the central area of *Nrf1*^*f/f*^*;Six3-Cre* retina. Scale bars: 50 μm in **a–d**, 100 μm in **e** and **f**. WT: wildtype

In E12.5 wildtype retinas, while the newly differentiated Isl1+ RGCs could be readily detected in the central retina (Fig. 3a), only a few Isl1+ cells were present in *Nrf1* mutant retinas (Fig. 3b). At E14.5, while differentiating Pou4f2+ RGCs were widespread across the neuroblast and ganglion cell layers in wildtype retinas (Fig. 3c), fewer Pou4f2+ RGCs were detected in Nrf1 mutant retinas (Fig. 3d). No clear ganglion cell layer could be seen in *Nrf1* mutant retinas. Furthermore, Pou4f2+ RGCs were distributed unevenly and formed patched clumps in the central region of the mutant retina (arrowheads in Fig. 3d). In E16.5 wildtype retinas, a distinct ganglion cell layer was formed, and newly differentiated Pou4f2+ RGCs were seen in the neuroblast layer (Fig. 3e). In contrast, a much thinner ganglion cell layer was observed in *Nrf1*^*f/f*^*;Six3-Cre* retinas, and Pou4f2+ RGCs were spread to the peripheral region (Fig. 3f). Together, these data suggest that RGC differentiation was delayed, and newly differentiated RGCs had defects in migrating toward the vitreous layer in the *Nrf1*^*f/f*^*;Six3-Cre* retinas.

**Fig. 4:**
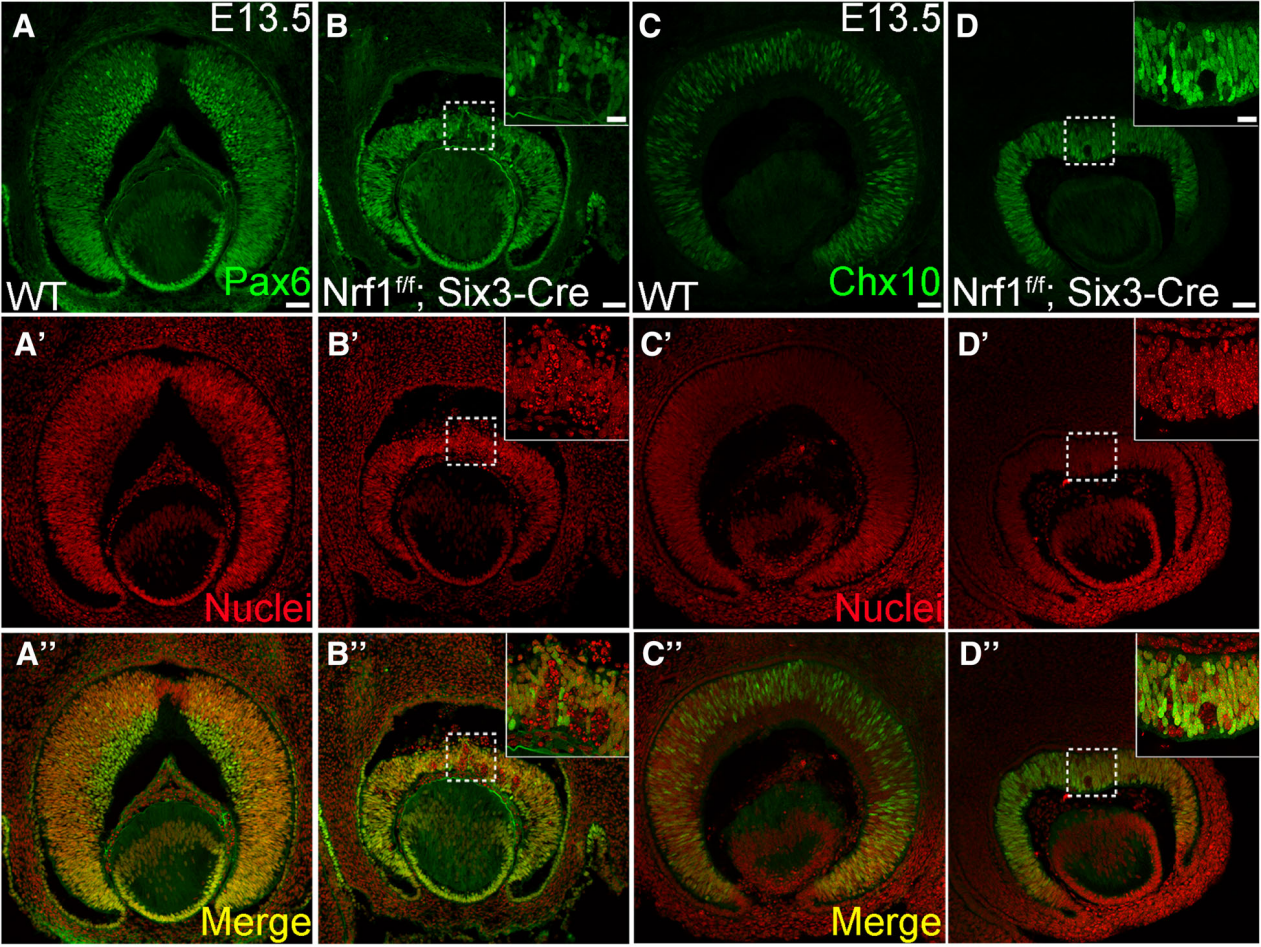
Expression of progenitor cell markers in wildtype and *Nrf1*^*f/f*^*;Six3-Cre* retina. (**a–d**) Immunostaining of E13.5 wildtype (**a**, **c**) and *Nrf1*^*f/f*^*;Six3-Cre* (**b**, **d**) retinal sections using anti-Pax6 antibody (**a**, **b**) and anti-Chx10 antibody (**c**, **d**). (**a**′–**d**′) Nuclei staining. (**a**′′–**d**′′) Merged images. Insets show higher magnification images of indicated areas. Scale bars: 50 μm. WT: wildtype

**Fig. 5:**
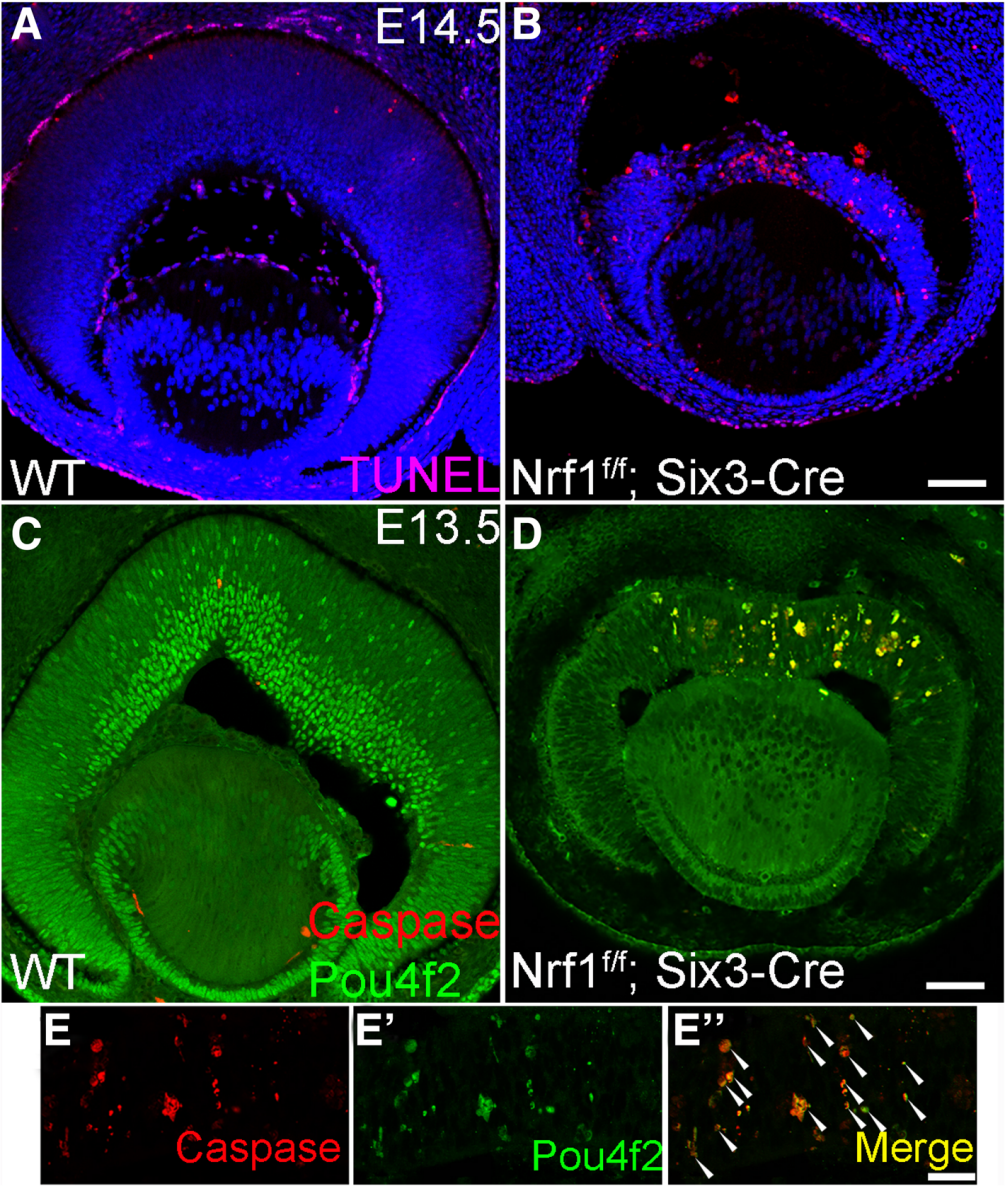
Differentiated RGCs undergo apoptosis in *Nrf1*^*f/f*^*;Six3-Cre* retina. (**a**, **b**) TUNEL assay on E14.5 wildtype (**a**) and *Nrf1*^*f/f*^*;Six3-Cre* (**b**) retinal sections. (**c**-**e**) E13.5 wildtype (**e**) and *Nrf1*^*f/f*^*;Six3-Cre* (**d**, **e**) sections labeled with anti-Pou4f2 and anti-cleaved caspase 3 antibodies. (**e**, **e**′ and **e**′′) Higher magnification images of *Nrf1*^*f/f*^*;Six3-Cre* sections labeled with anti-caspase (**e**) and Pou4f2 (**e**′) antibodies. (**e**′′) Merged images. Arrowheads indicate cells that are double positive for caspase and Pou4f2. Scale bars: 50 μm in **a** and **b**, 100 μm in **c** and **d**. WT: wildtype

To detect RPCs, wildtype and *Nrf1*^*f/f*^*;Six3-Cre* retinal sections were immunolabeled with anti-Pax6 or Chx10 antibodies (Fig. 4a–d). Pax6 and Chx10 were expressed in both wildtype and *Nrf1*^*f/f*^*;Six3-Cre* retinas. However, Pax6+ or Chx10+ RPCs were unevenly distributed in the central region of Nrf1^f/f^ retinas compared to the peripheral region (Fig. 4b, d). Interestingly, several clumps of RPCs lacking Pax6 expression were formed in Nrf1 mutant retinas, and the nuclei of these Pax6-negative cells appeared granulated, suggesting these were cells undergoing apoptosis (Fig. 4b, b’, b′′). Similarly, mis-patterned Chx10 expression and granular-shaped nuclei were observed in the central region of *Nrf1*^*f/f*^*;Six3-Cre* retinas (Fig. 4d, d’, d′′). Consistently, *Nrf1*^*f/f*^*;Six3-Cre* retinas contained significantly more apoptotic cells than wildtype retinas (Fig. 5a, b). The majority of apoptotic cells were found in the central region of *Nrf1*-mutant retinas. In addition, these apoptotic cells (marked by cleaved caspase 3 expression) were Pou4f2+ (Fig. 5c-e), indicating that ganglion cells had differentiated but could not migrate to the RGC layer and eventually died in situ.

### Severe reduction of proliferation in *Nrf1*^*f/f*^*;Six3-Cre* retina

**Fig. 6:**
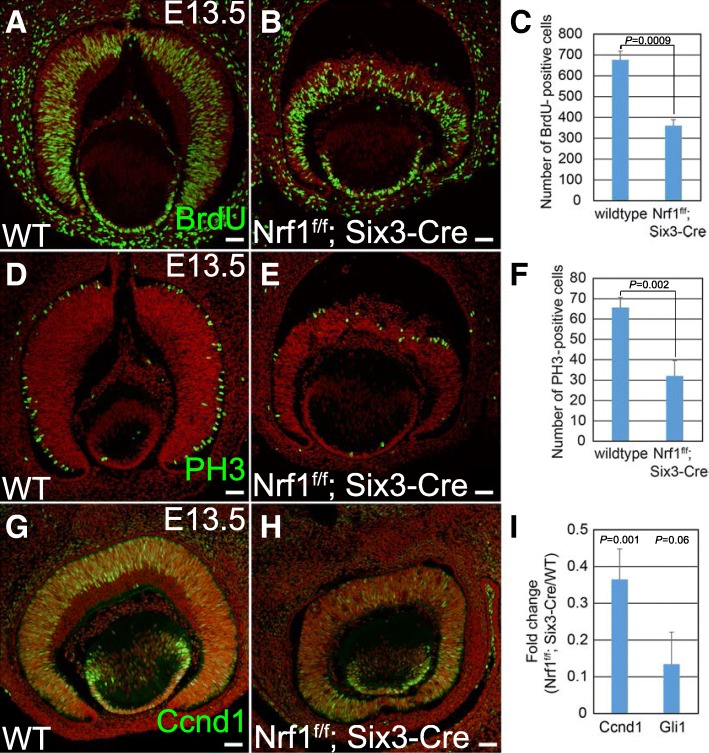
Reduced cell proliferation in *Nrf1*^*f/f*^*;Six3-Cre* retina. (**a**, **b**) BrdU labeling of E13.5 wildtype (**a**) and *Nrf1*^*f/f*^*;Six3-Cre* (**b**) retinal sections. (**c**) The number of BrdU+ cells in wildtype and *Nrf1*^*f/f*^*;Six3-Cre* sections. (**d**, **e**) E13.5 wildtype and *Nrf1*^*f/f*^*;Six3-Cre* retinal sections labeled with anti-PH3 antibody. (**f**) The number of PH3+ cells in wildtype and *Nrf1*^*f/f*^*;Six3-Cre* retinal sections. (**g**, **h**) Immunostaining of E13.5 wildtype and *Nrf1*^*f/f*^*;Six3-Cre* retinal sections with anti-Ccnd1 antibody. (**i**) qRT-PCR analysis of *Ccnd1* and *Gli1* in E13.5 WT and *Nrf1*^*f/f*^*;Six3-Cre* retinas. Scale 50 μm. WT: wildtype

The *Nrf1*^*f/f*^*;Six3-Cre* retina was substantially smaller and thinner than the wildtype retina, suggesting that the proliferation of RPCs in the *Nrf1*-mutant retina was compromised. To examine this phenotype, wildtype and *Nrf1*^*f/f*^*;Six3-Cre* retinas were immuno-labeled with several cell cycle markers. To detect S-phase proliferating RPCs, we pulse-labeled E13.5 embryos with BrdU and then conducted immunostaining using anti-BrdU antibody on retinal sections. We counted the number of BrdU+ cells in sections and found that the number of BrdU+ S-phase RPCs were reduced to ~ 50% in *Nrf1*^*f/f*^*;Six3-Cre* retinas compared to wildtype retinas (Fig. 6a-c, *P* = 0.0009). We then conducted immunostaining using anti-PH3 and anti-cyclin D1 (Ccnd1) antibodies on retinal sections to detect RPCs in M-phase and G1-phase, respectively. The number of PH3+ M-phase RPCs per section from *Nrf1*^*f/f*^*;Six3-Cre* was also reduced to ~ 50% compared to wildtype retinas (Fig. 6d-f, *P* = 0.002), and Ccnd1+ RPCs were nearly absent in the central region of *Nrf1*-mutant retinas (Fig. 6g, h). The PH3+ RPCs in the *Nrf1*-mutants were not properly positioned at the apical side as in control retinas (Fig. 6d, e). In addition, using qRT-PCR, we found that the expression levels of *Ccnd1* were reduced to ~ 10% in *Nrf1*-mutant retinas compared with wildtype retina, and *Gli1,* the key downstream effector of Shh pathway in RPCs [31, 32], was reduced to ~ 30% (Fig. 6i, *Ccnd1*: *P* = 0.001, *Gli1*: *P* = 0.006). Other Shh pathway genes, such as Shh and Ptch1, were also downregulated in *Nrf1*-mutant retinas (in GSE101550 dataset described in next section). Together, these data indicate that the RPC proliferation is reduced in *Nrf1*-mutant retinas.

### Identification of Nrf1-dependent retina-expressed genes at E13.5

**Fig. 7:**
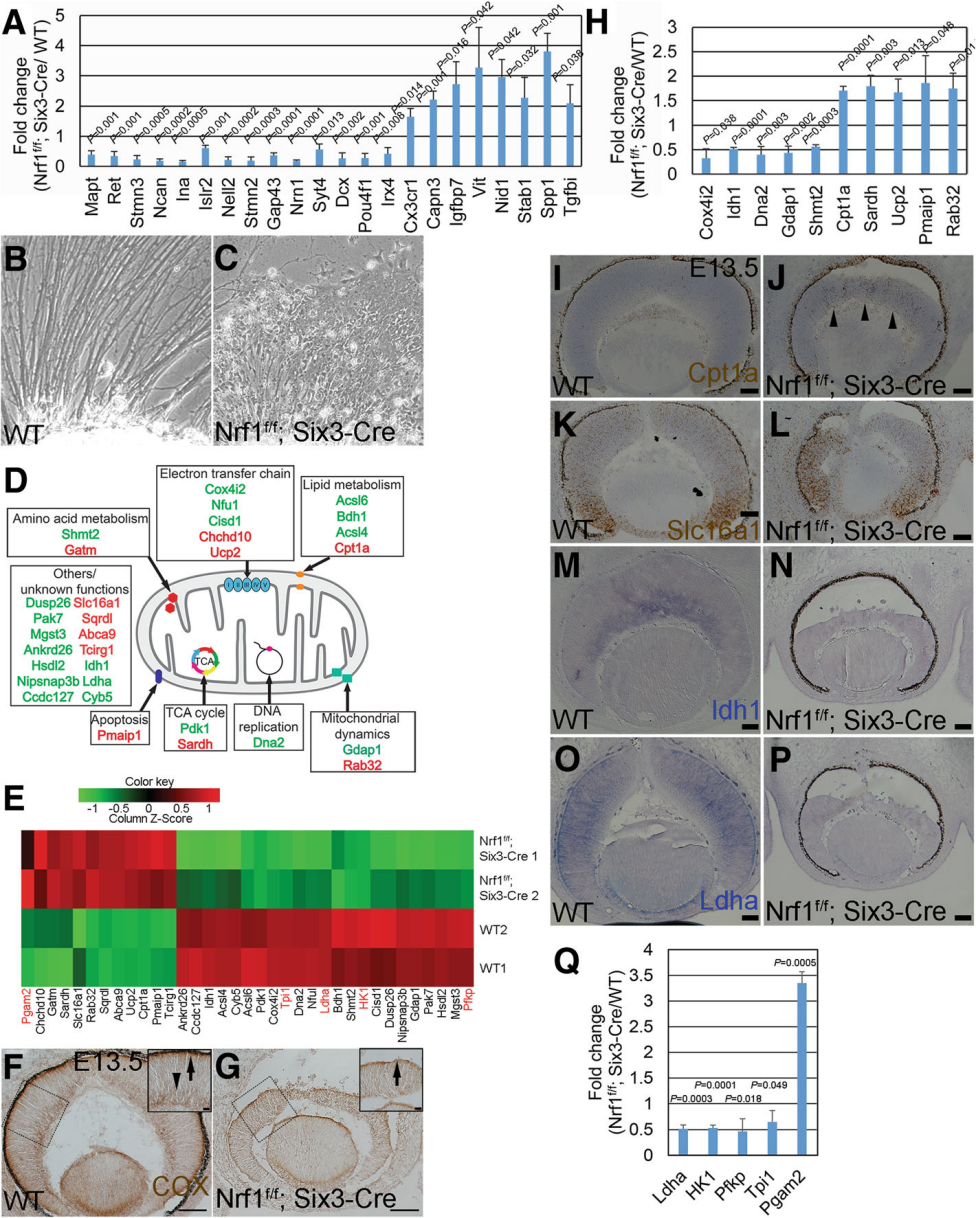
RNA-seq identifies genes involved in neurite outgrowth, mitochondrial functions, and energy production in *Nrf1*^*f/f*^*;Six3-Cre* retina. (**a**) qRT-PCR analysis of the 22 top affected genes identified in *Nrf1*^*f/f*^*;Six3-Cre* retinas, confirming changes in mRNA expression detected by RNA-seq. (**b**, **c**) Representative images of retinal explant cultures from E13.5 wildtype (**b**) and *Nrf1*^*f/f*^*;Six3-Cre* (**c**) embryos. (**d**) Schematic mapping of mitochondrial and functional annotation of upregulated (red) and downregulated (green) genes in *Nrf1*^*f/f*^*;Six3-Cre* retinas detected by RNA-seq. (**e**) Heatmap from *Nrf1*^*f/f*^*; Six3-Cre* RNA-seq showing 30 mitochondrial and 5 glycolytic genes whose expression changed. Mitochondrial genes are labeled in black, and glycolytic genes are labeled in red. (**f**, **g**) COX activity in E13.5 wildtype (**f**) and *Nrf1*^*f/f*^*;Six3-Cre* (**g**) retinas. Insets show higher magnification images of the indicated areas. Arrows indicate COX activities in the future photoreceptor layer; arrowheads indicate COX activities in ganglion cell layer. (**h**) qRT-PCR analysis of a subset of affected mitochondria genes between wildtype and *Nrf1*^*f/f*^*;Six3-Cre* retinas confirming changes of mRNA expression detected by RNA-seq. (**i**–**p**) In situ hybridization of mitochondrion-associated genes on E13.5 wildtype (**i**, **k**, **m**, and **o**) and *Nrf1*^*f/f*^*;Six3-Cre* (**j**, **l**, **n**, and **p**) retinal sections. Arrowheads indicate increased expression of *Cpt1a* in the central area of *Nrf1*^*f/f*^*;Six3-Cre* retina. (**q**) qRT-PCR analysis of glycolytic genes of wildtype and *Nrf1*^*f/f*^*;Six3-Cre* retinas. Scale bars: 100 μm in **f** and **g**, 20 μm in insets and 50 μm in **i–j**. WT: wildtype

To further investigate how Nrf1 regulates retinal development, we performed RNA-seq analysis on E13.5 wildtype and *Nrf1*^*f/f*^*;Six3-Cre* retinas to identify genes whose expression was affected in the *Nrf1*^*f/f*^*;Six3-Cre* retinas. The data discussed here have been deposited in NCBI’s Gene Expression Omnibus [33] and are accessible through GEO Series accession number GSE101550 (https://www.ncbi.nlm.nih.gov/geo/query/acc.cgi?acc=GSE101550). The analysis revealed 488 downregulated and 595 upregulated genes in *Nrf1*-mutant retinas compared to control retinas. We first conducted qRT-PCR analysis on the 22 most affected genes (14 down- and 8 up-regulated) and found that their relative expression levels between the E13.5 control and *Nrf1*-mutant retinas are consistent with the RNA-seq output, demonstrating the reliability of the RNA-seq data (Fig. 7a). Using gene ontology for biological process analysis (GO-BP) of these gene lists, the top 5 categories of the downregulated genes are genes involved in nervous system development, neurogenesis, neuron differentiation, generation of neurons, and neuron projection development (Table 2), and the upregulated genes are involved in cell adhesion, biological adhesion, regulation of cell projection, angiogenesis, and positive regulation of developmental process (Table 3).

**Table 2:** Top 10 GO terms relevant to 488 downregulated genes in E13.5 *Nrf1*^*f/f*^; *Six3-Cre* retinas

Rank	GO Category	GO ID	GO Term	Number of Focused Genes	*P* Value	FDR
1	GOTERM_BP_FAT	GO:0030182	nervous system development	156	1.70E-39	6.96E-36
2	GOTERM_BP_FAT	GO:0048666	neurogenesis	126	1.80E-35	3.80E-32
3	GOTERM_BP_FAT	GO:0031175	neuron differentiation	114	1.40E-34	2.00E-31
4	GOTERM_BP_FAT	GO:0048667	generation of neurons	119	8.00E-34	8.40E-31
5	GOTERM_BP_FAT	GO:0000904	neuron projection development	91	6.90E-33	5.80E-30
6	GOTERM_BP_FAT	GO:0048812	neuron development	99	7.00E-33	4.90E-30
7	GOTERM_BP_FAT	GO:0007409	neuron projection morphogenesis	68	5.00E-29	3.00E-26
8	GOTERM_BP_FAT	GO:0030030	cell morphogenesis involved in neuron differentiation	62	5.50E-26	2.90E-23
9	GOTERM_BP_FAT	GO:0019226	axonogenesis	55	2.70E-25	1.30E-22
10	GOTERM_BP_FAT	GO:0048858	cell projection organization	99	1.40E-24	5.90E-22
1	GOTERM_CC_FAT	GO:0030424	axon	54	4.00E-27	1.30E-24
2	GOTERM_CC_FAT	GO:0045202	synapse	57	5.30E-23	8.90E-21
3	GOTERM_CC_FAT	GO:0043025	neuronal cell body	58	1.40E-22	1.60E-20
4	GOTERM_CC_FAT	GO:0043005	neuron projection	51	5.70E-22	4.80E-20
5	GOTERM_CC_FAT	GO:0016020	membrane	243	9.40E-16	6.00E-14
6	GOTERM_CC_FAT	GO:0030425	dendrite	46	1.90E-15	1.10E-13
7	GOTERM_CC_FAT	GO:0010469	postsynaptic density	31	1.10E-14	1.70E-12
8	GOTERM_CC_FAT	GO:0030054	cell junction	53	1.80E-13	7.50E-12
9	GOTERM_CC_FAT	GO:0043195	terminal bouton	21	1.10E-12	4.10E-11
10	GOTERM_CC_FAT	GO:0030426	growth cone	24	1.80E-12	6.10E-11
1	GOTERM_MF_FAT	GO:0008092	cytoskeletal protein binding	52	2.40E-10	2.40E-07
2	GOTERM_MF_FAT	GO:0022836	gated channel activity	26	6.10E-08	2.70E-05
3	GOTERM_MF_FAT	GO:0005216	ion channel activity	29	2.60E-07	7.50E-05
4	GOTERM_MF_FAT	GO:0022838	substrate-specific channel activity	29	4.90E-07	1.10E-04
5	GOTERM_MF_FAT	GO:0015631	tubulin binding	23	7.60E-07	1.30E-04
6	GOTERM_MF_FAT	GO:0005261	cation channel activity	23	1.20E-06	1.80E-04
7	GOTERM_MF_FAT	GO:0022803	passive transmembrane transporter activity	29	2.00E-06	2.50E-04
8	GOTERM_MF_FAT	GO:0015267	channel activity	29	2.00E-06	2.50E-04
9	GOTERM_MF_FAT	GO:0019905	syntaxin binding	12	7.10E-06	7.80E-04
10	GOTERM_MF_FAT	GO:0017075	syntaxin-1 binding	7	1.10E-05	1.00E-03

*GO* gene ontology, *FDR* false discovery rate

**Table 3:** Top 10 GO terms relevant to 595 upregulated genes in E13.5 *Nrf1*^*f/f*^; *Six3-Cre* retinas

Rank	GO Category	GO ID	GO Term	Number of Focused Genes	*P* Value	FDR
1	GOTERM_BP_FAT	GO:0007155	cell adhesion	70	1.40E-22	5.00E-33
2	GOTERM_BP_FAT	GO:0022610	biological adhesion	70	1.60E-22	3.90E-33
3	GOTERM_BP_FAT	GO:0042127	regulation of cell projection	57	4.80E-15	3.70E-31
4	GOTERM_BP_FAT	GO:0001525	angiogenesis	23	2.40E-10	3.30E-31
5	GOTERM_BP_FAT	GO:0051094	positive regulation of developmental process	29	9.60E-10	7.80E-31
6	GOTERM_BP_FAT	GO:0007423	sensory organ development	31	1.00E-09	9.90E-31
7	GOTERM_BP_FAT	GO:0048514	blood vessel morphogenesis	27	1.20E-09	1.10E-29
8	GOTERM_BP_FAT	GO:0001568	blood vessel development	30	2.00E-09	1.10E-29
9	GOTERM_BP_FAT	GO:0009611	response to wounding	36	2.10E-09	1.20E-28
10	GOTERM_BP_FAT	GO:0001944	vasculature development	30	9.60E-09	1.20E-28
1	GOTERM_CC_FAT	GO:0031012	extracellular matrix	77	7.30E-29	4.10E-26
2	GOTERM_CC_FAT	GO:0005578	proteinaceous extracellular matrix	62	9.90E-27	2.80E-24
3	GOTERM_CC_FAT	GO:0044421	extracellular region part	231	9.50E-24	1.80E-21
4	GOTERM_CC_FAT	GO:0031982	membrane-bounded vehicle	206	5.60E-21	7.90E-19
5	GOTERM_CC_FAT	GO:0005576	extracellular region	245	9.40E-21	1.10E-18
6	GOTERM_CC_FAT	GO:0009986	cell surface	88	1.30E-20	1.20E-18
7	GOTERM_CC_FAT	GO:0044420	extracellular matrix component	33	4.50E-19	3.70E-17
8	GOTERM_CC_FAT	GO:1903561	extracellular vesicle	168	2.70E-18	1.90E-16
9	GOTERM_CC_FAT	GO:0043230	extracellular organelle	168	3.50E-18	2.20E-16
10	GOTERM_CC_FAT	GO:0070062	extracellular exosome	166	9.20E-18	5.20E-16
1	GOTERM_MF_FAT	GO:0005212	structural constituent of eye lens	16	9.60E-18	1.10E-17
2	GOTERM_MF_FAT	GO:0005515	protein biding	196	9.70E-14	1.60E-12
3	GOTERM_MF_FAT	GO:0005178	integrin binding	22	1.20E-12	1.80E-11
4	GOTERM_MF_FAT	GO:0005518	collagen binding	16	1.10E-10	4.50E-10
5	GOTERM_MF_FAT	GO:0005509	calcium ion binding	52	2.60E-09	5.00E-09
6	GOTERM_MF_FAT	GO:0005201	extracellular matrix structural constituent	12	1.90E-08	3.20E-08
7	GOTERM_MF_FAT	GO:0008201	heparin binding	21	2.10E-08	6.10E-08
8	GOTERM_MF_FAT	GO:0050840	extracellular matrix binding	10	6.80E-08	2.70E-07
9	GOTERM_MF_FAT	GO:0004872	receptor activity	21	1.90E-07	4.00E-07
10	GOTERM_MF_FAT	GO:0004714	transmembrane receptor protein tyrosine kinase activity	12	4.10E-07	6.10E-07

*GO* gene ontology, *FDR* false discovery rate

Since severe RGC loss was observed in *Nrf1*^*f/f*^*;Six3-Cre* retinas, we expected that RGC gene expression would be reduced in *Nrf1*-deficient retinas. Atoh7 is a key factor essential for RGC development. RNA-seq data revealed that *Atoh7* expression was slightly reduced by ~ 19.5% in *Nrf1*-mutant retinas; however, Atoh7-expressing precursor cells can be readily detected in *Nrf1*-mutant retinas (data not shown), suggesting that the RGC loss phenotype is mainly due to a defective RGC differentiation process. Transcriptome analysis comparing Atoh7+ RPCs and Atoh7-negative cells in E13.5 has revealed 236 genes with altered expression levels [34]. We compared the 488 genes that are downregulated in *Nrf1*^*f/f*^*;Six3-Cre* retinas with the 236 genes enriched in Atoh7+ RPCs and found 121 common genes (Table 4). The majority of these genes were expressed in RGCs, including *Pou4f1*, *Pou4f2*, *Isl1*, and *Myt1*, which are known to be expressed in differentiating RGCs [35]. In addition, 41 genes involved in neuronal differentiation were found, such as *neurofilament light chain* (*Nefl*) and *neurofilament middle chain* (*Nefm*). We also compared the 488 downregulated genes in *Nrf1*^*f/f*^*;Six3-Cre* to the 49 significantly downregulated genes in the Pou4f2^-/-^ retina [36] and found 18 common genes downregulated in both *Nrf1*^*f/f*^*;Six3-Cre* and *Pou4f2*^-/-^ retinas (data not shown). Among them, 7 genes are enriched in Atoh7+ retinas. These results indicate *Nrf1* depletion affects RGC gene expression.

**Table 4:** Genes downregulated in *Nrf1*^*f/f*^; *Six3-Cre* that are enriched in Atoh7+ cells

	Gene name	FC	Spatial expression
Transcription factor	Barhl2	−1.63	RGC
	Ebf1	−2.63	RGC
	*Ebf3*	−3.09	RGC
	Irx2	− 2.35	RGC
	Irx3	−1.97	RGC
	Irx5	−2.43	RGC
	Irx6	−2.18	RGC
	Isl1	−2.43	RGC
	Myt1	−2.14	RGC
	Onecut3	−2.15	RGC
	*Pou4f1*	−2.62	RGC
	*Pou4f2*	−1.89	RGC
	Pou6f2	−2.28	RGC
	Ptf1a	−2.02	retina
	Tub	−2.38	RGC
Neuron differentiation	Actl6b	−2.2	RGC
	Adcyap1	−1.71	RGC
	Bsn	−2.27	RGC
	Celsr3	−2.35	RGC
	Cend1	−1.89	RGC
	Cntn2	−3.09	RGC
	Dcx	−2.87	RGC
	Dner	−2.08	RGC
	Dnm3	−1.7	unknown
	Dok5	−1.52	retina
	Dscam	−2.5	retina
	Elavl3	−1.52	RGC
	Elavl4	−2.67	retina
	*Gap43*	−3.02	RGC
	Gprin1	−2.51	retina
	Ina	−3.56	RGC
	Insc	−1.88	unknown
	Islr2	−3.49	RGC
	Kif5a	−2.66	RGC
	Klhl1	−1.76	RGC
	L1cam	−2.65	RGC
	*Mapt*	−4.37	RGC
	Mmp24	−2.32	RGC
	Myo16	−1.61	RGC
	Myt1l	−2.61	RGC
	*Nefl*	−3.67	RGC
	Nefm	−2.46	RGC
	*Nell2*	−3.49	RGC
	Nptx1	−1.63	RGC
	Nrn1	−2.91	RGC
	Ret	−3.75	RGC
	Scn3b	−2.77	RGC
	Scrt1	−2.11	RGC
	Sez6l	−2.56	RGC
	Slit1	−1.81	RGC
	Snap25	−2.41	RGC
	Stmn2	−3.26	RGC
	Stmn3	−3.65	RGC
	Th	−2.45	retina
	Tnik	−1.63	unknown
	Tubb3	−2.38	RGC
	Unc13a	−2.62	RGC
Others	1810041L15Rik	−2.49	RGC
	A930011O12Rik	−2.08	unknown
	Ajap1	−1.66	RGC
	Akap6	−3.68	RGC
	Apba2	−1.93	RGC
	Arg1	−1.55	RGC
	Atp1a3	− 1.7	retina
	Cacna1b	−2.28	RGC
	Calb2	−2.96	RGC
	Ccnd1	−1.83	RPC
	Cda	−1.56	RGC
	Celf3	−2.19	RGC
	Celf5	−1.89	retina
	Chga	−1.65	RGC
	Chgb	−1.72	RGC
	Chst8	−1.95	RGC
	Coro2a	−1.85	RGC
	Crmp1	−2.78	RGC
	D930028M14Rik	−1.8	RGC
	Disp2	−2.82	RGC
	Dnajc6	−1.78	RGC
	Dusp26	−2.37	RGC
	Eya2	−1.59	RGC
	Fam155a	−1.76	RGC
	Fam78b	−1.73	unknown
	Fgf15	−1.61	RPC
	Gabbr2	−1.97	unknown
	Gdap1l1	−1.91	RGC
	Grm2	−2.67	unknown
	Hecw1	−2.5	RGC
	Hspa12a	−2.21	retina adult
	Igfbpl1	−2.25	RGC
	Iqsec3	−1.53	RGC
	Kcnq2	−2.47	unknown
	Mapk11	−1.75	RGC
	Mtus2	−2.7	RGC
	Nacad	−2.65	RGC
	Nhlh2	−2.68	RGC
	Nmnat2	−2.43	RGC
	Nsg2	−3.26	RGC
	Pak7	−2.04	unknown
	Ppp2r2b	−1.8	RGC
	Ppp2r2c	−1.88	unknown
	Rab3c	−2.12	RGC
	Rph3a	−2.58	RGC
	Rtn1	−2.59	RGC
	Rundc3a	−2.27	RGC
	Rusc1	−1.92	RGC
	Scg3	−2.94	RGC
	Scn3a	−2.4	unknown
	Sez6l2	−2.56	RGC
	Slc17a6	−2.88	RGC
	Smpd3	−1.97	RGC
	Sncg	−4.74	RGC
	Spire2	−1.54	RGC
	Srrm3	−2	unknown
	Sst	−2.14	RGC
	Stk32a	−1.83	RGC
	Svop	−2.17	RGC
	Thsd7b	−2.45	unknown
	Trim46	−1.56	unknown
	Trp53i11	−1.79	RGC
	Tubb2a	−2.35	unknown
	Unc79	−2.31	unknown
	Vwa5b2	−1.67	RGC
	Xkr7	−1.69	unknown

488 downregulated genes in *Nrf1*^*f/f*^; *Six3-Cre* retinas compared with 236 genes enriched in Atoh7+ retinal cells [34] identified 121 common genes. They are listed with official gene name, RNA-seq fold difference, and spatial expression pattern. Italicized gene names indicated downregulated genes in E14.5 Pou4f2^−/−^ retinas [35]. *FC* fold change

### Defective axon outgrowth in *Nrf1*^*f/f*^*;Six3-Cre* retinas

To determine whether retinal neurons were defective in neurite outgrowth, we cultured retinal explants from E13.5 wildtype and *Nrf1*^*f/f*^*;Six3-Cre* embryos to examine axonal outgrowth. Consistent with the RNA-seq analysis, we found that retinal explants from *Nrf1*^*f/f*^*;Six3-Cre* embryos failed to form and extend well-bundled axons as in wildtype explants (Fig. 7b, c), indicating an important function for Nrf1 in regulating genes involved in neurite outgrowth.

### Altered expression of genes associated with mitochondrial function and energy production in *Nrf1*^*f/f*^*;Six3-Cre* retinas

Because Nrf1 is a key regulator of nuclear-encoded genes involved in mitochondrial functions, we then tested whether genes involved in mitochondrial functions were altered in *Nrf1*-deficient retinas. By comparing the gene list in MitoCarta 2.0 [37, 38], we revealed a subset of genes with altered expression levels in *Nrf1*-mutant retinas, and color-coded and mapped them to various functional subdomains in the mitochondria (Fig. 7d). In addition, 5 glycolysis genes with affected expression levels in *Nrf1*-deficient retinas were identified (Fig. 7e, q). For example, mRNA levels of *cytochrome c oxidase subunit 4i2* (*Cox4i2*) in *Nrf1*-mutant retinas were reduced to ~ 44% of those in wildtype retinas. We tested mitochondrial respiratory activity in *Nrf1*^*f/f*^*;Six3-Cre* retinas by examining the histochemical activity of COX. Intense COX activity was detected in RGCs (arrowhead in Fig. 7f) and the outermost area of retina where photoreceptor precursors resided (arrow in Fig. 7f). In contrast, COX activity was diminished to background levels in the *Nrf1*^*f/f*^*;Six3-Cre* retina (Fig. 7g). We then performed qRT-PCR analysis on a small, selected set of these affected genes and found that the levels of expression of all of them were consistent with the RNA-seq data (Fig. 7h, *Cox4i2*: *P* = 0.038, *Idh*: *P* = 0.0001, *Dna2*: *P* = 0.003, *Gdap1*: *P* = 0.002, *Shmt2*: *P* = 0.003, *Cpt1a*: *P* = 0.0001, *Sardh*: *P* = 0.003, *Ucp2*: *P* = 0.013, *Pmaip1*: *P* = 0.048, *Rab32*: *P* = 0.014).

Furthermore, we performed in situ hybridization (ISH) for several genes whose expression was either upregulated (*Cpt1a* and *Slc16a1*) or downregulated (*Idh1*, *Ldha*) in *Nrf1* mutants by RNA-seq analysis. *Cpt1a*, encoding carnitine palmitoyltransferase 1a, is involved in lipid transfer in mitochondria. In E13.5 wildtype retinas, *Cpt1a* was expressed at extremely low levels, barely detectable even by ultrasensitive RNAscope ISH (Fig. 7i). In *Nrf1*-mutant retinas, weak but detectable *Cpta1* transcripts were visible in the central retina (arrowheads in Fig. 7j). *Slc16a1*, encoding solute carrier family 16 (monocarboxylic acid transporters) member 1, is involved in lactate/pyruvate transport in mitochondria. *Slc16a1* was expressed in the peripheral retina in E13.5 wildtype retinas and upregulated in the central area of *Nrf1*^*f/f*^*;Six3-Cre* retinas (Fig. 7k, l). *Idh1*, encoding isocitrate dehydrogenase 1, was highly expressed in RGCs in wildtype retinas, whereas its expression was drastically reduced in *Nrf1*^*f/f*^*;Six3-Cre* retinas (Fig. 7m, n). *Ldha*, encoding lactate dehydrogenase A, which catalyzes the conversion of lactate to pyruvate in the glycolysis pathway, was highly expressed in RPCs in wildtype retinas but downregulated in *Nrf1*^*f/f*^*;Six3-Cre* retinas (Fig. 7o, p). To confirm the effect of *Nrf1* deletion on the 5 genes involved in the glycolysis pathway, we performed qRT-PCR analysis for these 5 glycolysis-associated genes (Fig. 7q, *Ldha*: *P* = 0.0003, *HK1*: *P* = 0.0001, *Pfkp*: *P* = 0.018, *Tpi1*: *P* = 0.049, *Pgam2*: *P* = 0.0005) and confirmed that expression levels of these genes were indeed affected as revealed by RNA-seq analysis. These data indicate that Nrf1 is important in regulating various metabolic pathways, including lipid metabolism, glycolysis, and oxidative phosphorylation, during retinal development.

### Deleting *Nrf1* in rod photoreceptors caused complete rod degeneration

**Fig. 8:**
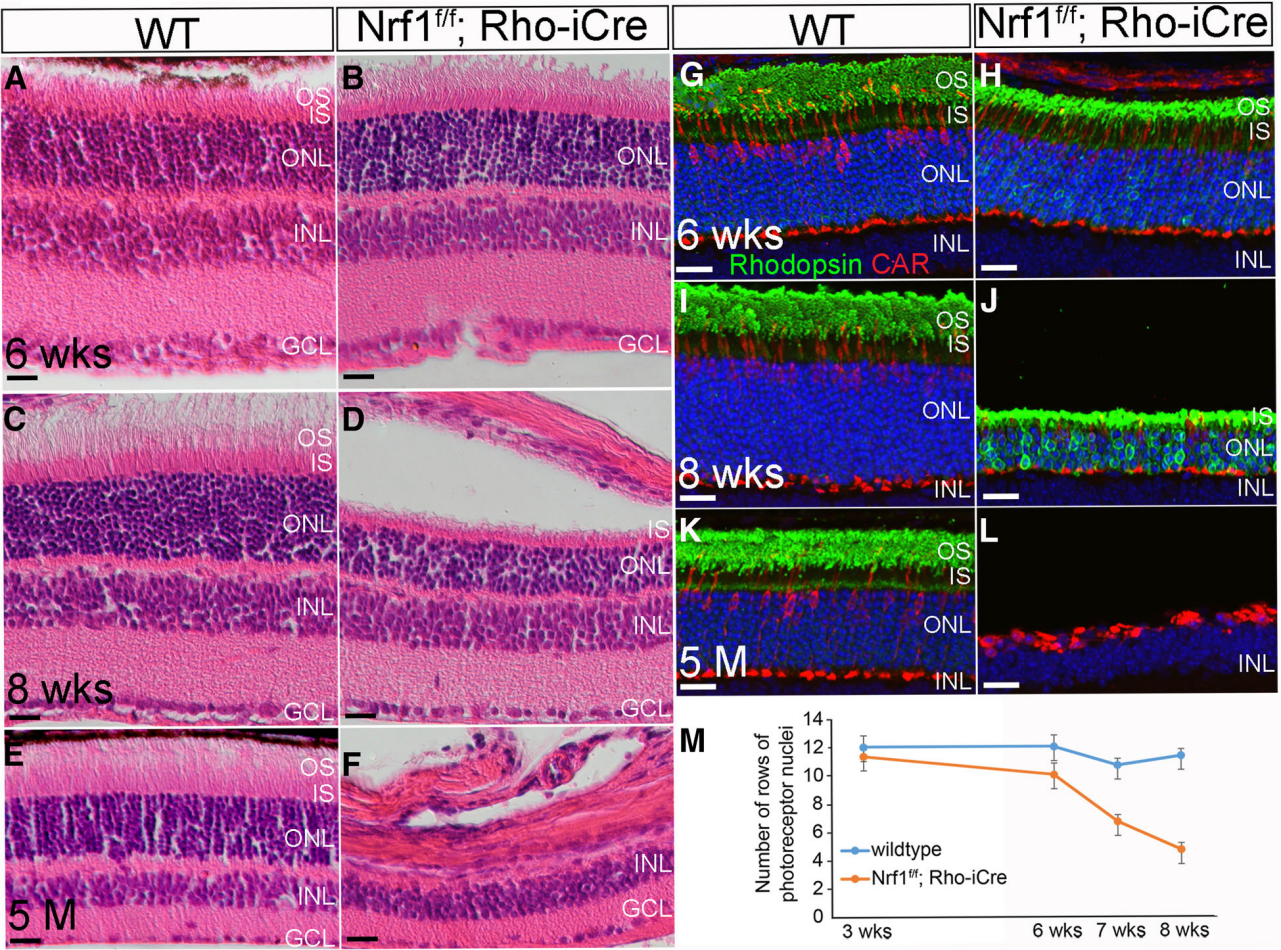
*Nrf1* conditional knockout by *Rho-iCre* causes severe photoreceptor degeneration. (**a–f**) H&E staining of retinal sections from wildtype (**a**, **c**, and **e**) and *Nrf1*^*f/f*^*;Rho-iCre* (**b**, **d**, and **f**) at 6 weeks, 8 weeks, and 5 months old. (**g–l**) Immunostaining of wildtype (**g**, **i**, and **k**) and *Nrf1*^*f/f*^*;Six3-Cre* (**h**, **j**, and **l**) retinal sections with anti-rhodopsin and anti-cone arrestin antibodies at 6 weeks, 8 weeks, and 5 months old. (**m**) The number of rows of photoreceptor nuclei in wildtype and *Nrf1*^*f/f*^*;Rho-iCre*. Scale bars: 20 μm. OS: outer segment. IS: inner segment. ONL: outer nuclear layer. INL: inner nuclear layer. GCL: ganglion cell layer. WT: wildtype

To investigate the in vivo function of *Nrf1* in differentiated neurons, we choose to use rod PRs as a model system, because rod PRs are the major neuronal type in the retina, and a large number of genetic mutations causing PR degeneration have been identified [39]. We bred a *Rho-iCre* transgenic mouse line with mice harboring *Nrf1*^*flox*^ allele to delete *Nrf1* in the photoreceptor cells. Prior to 6 weeks of age, *Nrf1*^*f/f*^*;Rho-iCre* retinas did not show any sign of histological phenotype compared with wildtype retinas (Fig. 8a, b). Starting from 8 weeks, the thickness of the ONLs in the *Nrf1*^*f/f*^*;Rho-iCre* retinas was notably thinner than that in the control retinas (Fig. 8c, d), and the number of PRs decreased to 50% of that in wildtype retinas (Fig. 8m, 3 weeks: *P* = 0.373, 6 weeks: *P* = 0.070, 7 weeks: *P* = 0.001, 8 weeks: *P* = 0.0001). At 5 months, the ONLs had almost disappeared (Fig. 8e, f).

To determine whether cone photoreceptors were also affected in rod-*Nrf1*-mutants, we immunolabeled wildtype and *Nrf1*^*f/f*^*;Rho-iCre* retinas with rod-specific rhodopsin and cone-specific arrestin (CAR). At 6 weeks of age, rhodopsin was enriched in the outer segments (OSs) of rod PRs. We observed slightly upregulated rhodopsin in *Nrf1*^*f/f*^*;Rho-iCre* retinas compared to wildtype retinas. There was no difference in numbers of CAR+ cells between control and mutant retinas. OSs and ISs were shorter in *Nrf1*^*f/f*^*;Rho-iCre* retinas than in wildtype retinas (Fig. 8g, h). In 8-week-old retinas, rhodopsin+ PRs in *Nrf1*^*f/f*^*;Rho-iCre* retinas were reduced to ~ 30% of wildtype retinas, while strong rhodopsin staining was detected in ONLs of *Nrf1*^*f/f*^*;Rho-iCre* retinas (Fig. 8i, j). At this stage, the number of cone photoreceptor cells was also reduced in *Nrf1*^*f/f*^*;Rho-iCre* retinas compared with wildtype retinas (Fig. 8i, j). In 5-month-old mutant retinas, no rhodopsin+ PRs were detected, while the few remaining cone photoreceptors formed a single column in the ONLs without the distinguishable normal cone-shaped morphology (Fig. 8k, l). Since rod PRs are required for cone survival [40, 41], the cone degeneration in *Nrf1*^*f/f*^*;Rho-iCre* retinas was likely secondary to rod PR degeneration. These results clearly indicate that *Nrf1* is essential for the survival of photoreceptor cells.

### Abnormal mitochondrial morphology and impaired mitochondrial functions in *Nrf1*^*f/f*^*;Rho-iCre* inner segments

**Fig. 9:**
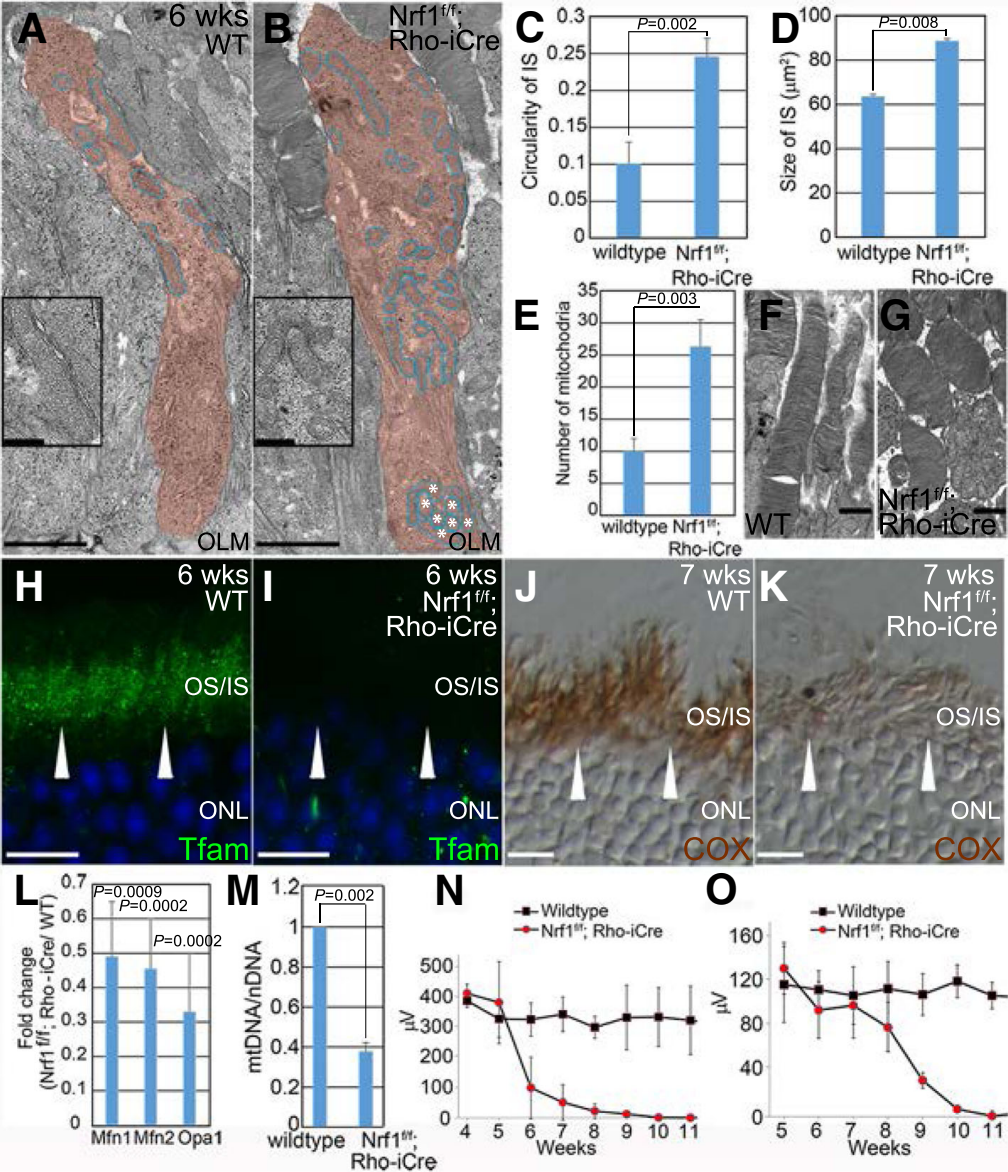
Defective mitochondria and ERG response in *Nrf1*^*f/f*^*;Rho-iCre* retina. (**a**, **b**) Transmission electron microscopy (TEM) images of the inner segments of wildtype and *Nrf1*^*f/f*^*;Rho-iCre* photoreceptors. Inner segment is color-labeled in red, and mitochondria are circled in blue. Asterisks indicate clustered mitochondria near the OLM in *Nrf1*^*f/f*^*;Rho-iCre* ISs. Insets show higher magnification images of indicated areas. (**c**–**e**) TEM images were analyzed with Fiji for the circularity of ISs (**c**), size of ISs (**d**) and the number of mitochondria (**e**). **f**, **g** TEM images of the outer segments of wildtype (**f**) and *Nrf1*^*f/f*^*;Rho-iCre* (**g**) photoreceptors. (**h**, **i**) Immunostaining of 6-week wildtype (**h**) and *Nrf1*^*f/f*^*;Rho-iCre* (**i**) retinal sections with anti-Tfam antibody. Arrowheads indicate Tfam staining in ISs. (**j**, **k**) COX activity of 7-week wildtype (**j**) and *Nrf1*^*f/f*^*;Rho-iCre* (**k**) retinal sections. Arrowheads indicate COX activities in ISs. (**l**) qRT-PCR analysis of genes involved in mitochondria fusion of wildtype and *Nrf1*^*f/f*^*;Rho-iCre* retinas. (**m**) Mitochondria copy number of 6-week-old wildtype and *Nrf1*^*f/f*^*;Rho-iCre* retinas. (**n**, **o**) ERGs of wildtype and *Nrf1*^*f/f*^*;Rho-iCre* littermates under dark-adapted (scotopic, **n**) and light-adapted (photopic, **o**) conditions. Scale bars: 1 μm in **a**, **b**, **f** and **g**, 10 μm in **h–k**. OLM: outer limiting membrane. OS: outer segment. IS: inner segment. ONL: outer nuclear layer. WT: wildtype

To examine how *Nrf1* deletion affected mitochondria in rod PRs, we used transmission electron microscopy to inspect the morphology of mitochondria in 6-week-old *Nrf1*^*f/f*^*;Rho-iCre* retinas, when the ISs had not yet degenerated. We collected transmission electron microscopy images of ISs from wildtype *Nrf1*^*f/f*^*;Rho-iCre* photoreceptors and analyzed with Fiji for the circularity of IS, size of IS, and number of mitochondria. We found that the ISs in *Nrf1*^*f/f*^*;Rho-iCre* retinas were slightly wider than the wildtype ISs (Fig. 9a-c, *P* = 0.002), resulting in a ~ 40% increase in size compared to wildtype retinas (Fig. 9d, *P* = 0.008). The number of mitochondria in a *Nrf1*^*f/f*^*;Rho-iCre* IS section was 2.5 times than that of wildtype (Fig. 9e, *P* = 0.003). The mitochondria in the *Nrf1*^*f/f*^*;Rho-iCre* ISs were notably smaller and displayed a more rounded shape compared to mitochondria in the control retinas and were more widely distributed within the ISs (Fig. 9a, b). A cluster of mitochondria was observed near the outer limiting membranes while no mitochondria were present in the same area in the wildtype ISs (asterisks in Fig. 9b). We also noticed that the OSs in *Nrf1*^*f/f*^*;Rho-iCre* photoreceptors were shorter than that of the controls (Fig. 9f, g).

Nuclear-encoded mitochondrial transcription factor A (Tfam/mtTFA), a key regulator of mitochondrial transcription and mitochondrial genome replication, is a known downstream target of Nrf1 [42]. To examine whether Tfam was affected in Nrf1-deficient rod PRs, we inspected Tfam expression by immunostaining and found that Tfam expression was abolished in *Nrf1*^*f/f*^*;Rho-iCre* ISs whereas strong expression of Tfam was observed in the wildtype ISs (Fig. 9h, i). Abnormalities in the number, morphology, and distribution of mitochondria, and the downregulation of a key mitochondrial regulator Tfam in *Nrf1*^*f/f*^*;Rho-iCre* ISs prompted us to determine whether mitochondrial function was compromised. We performed a COX assay to examine the mitochondrial enzymatic activity. As expected, COX activity was weaker in *Nrf1*^*f/f*^*;Rho-iCre* ISs compared with wildtype ISs (Fig. 9j, k). Furthermore, we tested whether the expression levels of genes involved in mitochondrial fusion were affected in *Nrf1*^*f/f*^*;Rho-iCre* retinas. Mitofusion-1 (Mfn1), Mfn2, and Optic Atrophy 1 (Opa1) are key mitochondrial proteins mediating mitochondrial fusion [43–45]. Deletion of *Mfn1* and *Mfn2* in skeletal muscle results in reduction of mtDNA and respiratory deficiencies [25]. We performed qRT-PCR to compare mRNA expression levels of *Mfn1*, *Mfn2,* and *Opa1* in 6-week-old wildtype and *Nrf1*^*f/f*^*;Rho-iCre* retinas. In *Nrf1*^*f/f*^*;Rho-iCre* retinas, *Mfn1*, *Mfn2,* and *Opa1* levels decreased to ~ 50% of wildtype retinas (Fig. 9l, *Mfn1*: *P* = 0.0009, *Mfn2*: *P* = 0.0002, *Opa1*: *P* = 0.0002). In addition, the copy numbers of mtDNA in *Nrf1*^*f/f*^*;Rho-iCre* retinas was ~ 38% compared to that of wildtype retinas (Fig. 9m), consistent with Tfam’s role as a major regulator of mtDNA replication and mitochondrial transcription.

Because *Nrf1*^*f/f*^*;Rho-iCre* retinas displayed severe rod degeneration followed by cone degeneration, we set out to track outer retina function using electroretinography (ERG). Dark-adapted wildtype and *Nrf1*^*f/f*^*;Rho-iCre* mice were exposed to calibrated light flashes for ERG recording. The scotopic a-wave amplitudes of *Nrf1*^*f/f*^*;Rho-iCre* mice were similar to those of wildtype before 5 weeks of age, began to decline at 6 weeks, and had completely diminished by 3 months (Fig. 9n, 4 weeks: *P* = 0.3101, 5 weeks: *P* = 0.4548. Six weeks: *P* = 1.6988E-05, 7 weeks: *P* = 3.0756E-09, 8 weeks: *P* = 9.7899E-12, 9 weeks: *P* = 2.7743E-16, 10 weeks: *P* = 9.8167E-05, 11 weeks: *P* = 0.00497). Photopic ERG b-wave amplitudes from light-adapted wildtype and *Nrf1*^*f/f*^*;Rho-iCre* mice were similar before 7 weeks of age, started to decline noticeably at 8 weeks, and were undetectable beyond 10 weeks (Fig. 9o, 5 week: *P* = 0.7052, 6 weeks: *P* = 0.2420, 7 weeks: *P* = 0.4169, 8 weeks: *P* = 0.0522, 9 weeks: *P* = 8.0496E-05, 10 weeks: *P* = 0.0002, 11 weeks: *P* = 6.8768E-05). These data indicate that PR functional loss precedes morphological defects and further demonstrate that deleting *Nrf1* in rod PRs causes abnormal mitochondria and impaired mitochondrial function, resulting in reduced outer retina activity and eventual complete photoreceptor loss.

## Discussion

Functional mitochondrial biogenesis is essential for energy metabolism, calcium homeostasis, the biosynthesis of amino acids, cholesterol, and phospholipids, elimination of excessive reactive oxygen species, and apoptosis. Nrf1 was identified as a major transcriptional regulator that connects the regulation of nuclear-encoded genes and mitochondrial biogenesis and has been implicated in the pathology of several neurodegenerative diseases [16, 46]. However, little is known about its role in central nervous system development because of the lack of an appropriate animal model. To fill this gap of knowledge, we generated *Nrf1* conditional knockout mouse models and used these mouse lines to conduct the first comprehensive in vivo study to delineate various roles of Nrf1 in proliferating neural progenitor cells, newly differentiated RGCs, and terminally differentiated rod PRs.

Previous studies have provided evidence for *Nrf1*’s role in cell growth and proliferation. For example, a genome-wide ChIP-chip study has revealed that Nrf1 binds and regulates a number of E2F-targeted genes involved in DNA replication and repair, mitosis and chromosome dynamics, and metabolism [47]. A ChIP-seq study using SK-N-SH human neuroblastoma cells has revealed that Nrf1 target genes contain genes associated with cell cycle regulation [16]. Cyclin D1-dependent kinase phosphorylates Nrf1 and inhibits its transcriptional activity [48]. *Nrf1*-deleted mouse embryos die during the peri-implantation stage between embryonic days 3.5 and 6.5 in part due to reduced cell proliferation [20]. In our study, we showed that deleting *Nrf1* in the proliferating RPCs reduced cell proliferation indices in the developing retina. The few surviving RPCs that exited the cell cycle and differentiated into RGCs failed to migrate from the neuroblast layer to the ganglion cell layer. Using RNA-seq analysis, we discovered that genes involved in neurite outgrowth are significantly downregulated in *Nrf1*-deficient retinas. Consistent with the RNA-seq data, we demonstrated that neurite outgrowth activity was reduced in *Nrf1*-deleted retinal explants compared to control explants. Although we cannot exclude the possibility that the RGC migration and neurite outgrowth phenotypes seen in *Nrf1*-mutants are caused indirectly by defective mitochondria, a recent study on a RPC-specific knockout of Ronin, a key transcriptional regulator for mitochondrial gene expression and RPC proliferation, has shown that conditionally deleting Ronin in RPCs causes defective mitochondrial function and premature cell cycle exit in RPCs, leading to the generation of more RGCs [49]. Interestingly, these extra, newly differentiated RGCs survive and do not display any defects as observed in *Nrf1*-mutants, suggesting that Nrf1 directly regulates subsets of genes for RGC migration and neurite outgrowth during retinal development. Together this in vivo and ex vivo evidence supports the previous findings that *Nrf1* is essential for cell growth, proliferation, and neurite outgrowth [50].

In the developing mouse retina, the proliferating RPCs and the terminally differentiated retinal neurons adopt different metabolic pathways for energy production. In RPCs, aerobic glycolysis is a predominant way to produce ATP, whereas oxidative phosphorylation is utilized in differentiated neurons [51]. Such a transition is observed in many developmental systems, suggesting that the reconfiguration of energy metabolic pathways is likely intricately mapped onto the regulatory networks controlling cell cycle progression and differentiation. In *Nrf1*-mutant retinas, *Ldha*, which encodes the enzyme that converts pyruvate to lactate and generates the nicotinamide adenine dinucleotide (NAD+) necessary for aerobic glycolysis [52], was significantly downregulated. Additionally, several glycolytic pathway genes were also downregulated in *Nrf1*-mutant RPCs, suggesting that *Nrf1*-mutant RPCs may shift to utilize oxidative phosphorylation to produce energy. Consistent with this, pyruvate dehydrogenase kinase isoenzyme 1 (Pdk1), a metabolic checkpoint enzyme that inactivates pyruvate dehydrogenase, was also downregulated in *Nrf1* mutant RPCs. Hence the increased pyruvate dehydrogenase activity would enable pyruvate to enter the tricarboxylic acid cycle. Despite Nrf1’s known function as a transcriptional activator, a subset of genes carrying out various mitochondrial functions, and *Pgam2*, encoding phosphoglycerate mutase which is involved in glycolysis, are upregulated in *Nrf1*-mutant retinas. Among them we observed the upregulation of *Cpt1a* and *Slc16a1* in mutant RPCs. It is currently unknown whether Nrf1 functions as a repressor that directly modulates the transcriptional levels of these genes or if deleting *Nrf1* indirectly leads to reprogramming in their transcriptional regulatory regions in RPCs. Nevertheless, these data taken together implicate Nrf1 in a regulatory role to enable RPCs to alter their metabolic program and advance to a committed neuronal fate. Although the molecular mechanism regulating the metabolic transition is currently unclear, the potential roles of metabolites in epigenetic control at several levels, including DNA methylation/demethylation and histone modifications, could influence the cellular state and fate [53]. Interestingly, a recent study showed that in vivo Nrf1 binding to its target sites is inhibited by de novo DNA methylation, and active demethylation and obstruction of de novo methylation through the binding of methylation-insensitive transcription factors could de-methylate the nearby genome, thus restoring Nrf1 binding and transcriptional activity [54]. Future research is required to uncover and compare the in vivo occupancy of Nrf1 and the methylome in proliferating RPCs and differentiated rod photoreceptors to determine whether this novel mechanism is actively utilized by Nrf1 and co-regulators in regulating metabolic transition.

The discovery of nuclear-encoded mitochondrial transcription factor A (Tfam/mtTFA) as a target of Nrf1 established the regulatory link between nuclear and mitochondrial gene expression [42]. In wildtype retinal photoreceptors, Tfam was transported to and enriched in the ISs, but its expression was undetectable in 6-week-old *Nrf1*-mutant ISs, confirming that *Tfam* is a bona fide in vivo target of Nrf1. The small, rounded mitochondrial morphology and the increased number of mitochondria seen in the ISs in *Nrf1*-deficient rods suggest that the normal mitochondrial fusion/fission processes are defective in *Nrf1*-mutant rods. Continuous mitochondrial fusion and fission are essential for maintaining a functional mitochondrial network to ensure sufficient exchange of mitochondrial contents, which might be otherwise damaged under stressed environments [55, 56]. Several key molecular regulators for mitochondrial fusion, including *Mfn1*, *Mfn2*, and *Opa1,* were downregulated in 6-week-old *Nrf1*-deficient rods. Because loss of any of these genes causes defects in mitochondrial fusion, impairs mitochondrial oxidative phosphorylation, and eventually leads to apoptosis, it is likely that defective mitochondrial fusion in *Nrf1*-null rods is a major cause of rod degeneration. Consistent with this, *ewg*, the Drosophila homolog of *Nrf1,* has been shown to play a role in regulating mitochondrial fusion and expression of the *Opa1*-like gene during muscle growth in the fly [57]. It is noteworthy that mutations in human *OPA1*, a direct target of human NRF1, are the cause of autosomal dominant optic atrophy [58], which leads to retinal ganglion cell death. Thus, it would be interesting to test whether downregulation of *Nrf1* contributes to RGC death in several glaucoma animal models.

For mammals with vascular retinas, mitochondria in the rod PRs migrate toward and localize in the outer part of the IS (the ellipsoid) for oxygen supplied from choriocapillaris [59, 60]. In *Nrf1*-null rods, however, mitochondria were often trapped near the base of the outer limiting membrane. Proper mitochondrial trafficking within a neuron is critical for clearing the older, damaged components and delivering the new materials encoded by nuclear genes [61]. It is therefore conceivable that mitochondrial trafficking defects in *Nrf1*-mutant retinas also contribute to the death of rod PRs.

Many mouse models of inherited retinal degenerative disease have been established to understand disease mechanisms and design treatment strategies for human diseases [62, 63]. In our study, we showed that *Rho-iCre* efficiently and specifically deleted *Nrf1* in rod cells as early as P10; however, the *Nrf1*-deficient rods degenerated at a relatively slow pace. By 4 weeks of age, we did not find histological differences between the controls and mutants. The first sign of degeneration in rod-Nrf1 mutants was the slight thinning of the ONLs and OSs and the reduction of the scotopic a-wave amplitudes. It took approximately 3 months for the *Nrf1*-deficient rods to completely degenerate. The reason for such resiliency is currently unknown. It is possible that the glycolysis pathway partially supports the energy demand in *Nrf1*-deficient rods. Alternatively, other transcriptional factors and epigenetic memory may transiently compensate for the loss of *Nrf1* to maintain the expression of *Nrf1*-regulated downstream genes. Nevertheless, the slow, progressive rod degeneration found in this new mouse model offers a unique opportunity to investigate how defective mitochondrial biogenesis affects different cellular processes whose defects frequently link to retinal degeneration. Furthermore, mitochondrial function declines with age and is associated with age-related disorders and cell death. It is of interest to test whether any of components in the Nrf1-regulated mitochondrial biogenesis pathway are associated with aging retinas and whether they can be used as therapeutic targets for ameliorating retinal degenerative diseases.

## Conclusions

Our findings confirm some of the known functions of Nrf1 that were previously revealed mainly through in vitro studies. Additionally, we uncovered a novel role for Nrf1 in metabolic reprogramming, although the degree to which Nrf1 is involved in this process during neural development remains to be determined. Our data also shed new light on how dysfunctional mitochondrial biogenesis may be involved in various neurodegenerative diseases. For example, we have shown that RPCs and newly differentiated RGCs are very sensitive to *Nrf1* deletion. In contrast, rod PRs, an energy demanding neuronal type, are much more tolerant of *Nrf1* deletion. We also found that the terminally differentiated RGCs are less sensitive to *Nrf1* deletion (data not shown). This difference may be in part due to the varying roles of *Nrf1* in different cell types and developmental stages; however, it also suggests that different neuronal tissues and cell lineages may have diverse sensitivities to mitochondrial defects. Future experiments using tissue- and cell-specific *Nrf1* deletions will be critical in directly addressing how dysfunctional mitochondrial biogenesis contributes to the pathology and disease progression in neurodegenerative diseases.

## Abbreviations

CAR: Cone-specific arrestin
Ccnd1: Cyclin D1
ChIP-seq: Chromatin immunoprecipitation sequencing
COX: Cytochrome c oxidase
ERG: Electroretinography
ES: Embryonic stem
ewg: Erect wing
FRT: FLP recombinase target
GCL: Ganglion cell layer
INL: Inner nuclear layer
IS: Inner segment
ISH: In situ hybridization
Mfn1: Mitofusion-1
Mfn2: Mitofusion-2
mtDNA: Mitochondrial DNA
nefl: Neurofilament light chain
nefm: Neurofilament middle chain
Nrf1: Nuclear respiratory factor 1
Nrf2/GABP: Nuclear respirator factor 2
OCT: Optimal cutting temperature
OLM: Outer limiting membrane
ONL: Outer nuclear layer
Opa1: Optic atrophy 1
OS: Outer segments
PARP-1: poly(ADP-ribose) polymerase 1
PBS: Phosphate buffered saline
PGC-1: Peroxisome proliferative activated receptor gamma coactivator 1
PR: Photoreceptor
qRT-PCR: Quantitative reverse transcriptase PCR
RGC: Retinal ganglion cell
RNA-seq: RNA sequencing
RPC: Retinal progenitor cell
TEM: Transmission electron microscopy
Tfam: Mitochondrial transcription factor A
TUNEL: Transferase dUTP nick end label
WT: Wildtype

## References

[1] Wu Z, Puigserver P, Andersson U, Zhang C, Adelmant G, Mootha V, Troy A, Cinti S, Lowell B, Scarpulla RC, Spiegelman BM (1999). Mechanisms controlling mitochondrial biogenesis and respiration through the thermogenic coactivator PGC-1. Cell.

[2] Virbasius CA, Virbasius JV, Scarpulla RC (1993). NRF-1, an activator involved in nuclear-mitochondrial interactions, utilizes a new DNA-binding domain conserved in a family of developmental regulators. Genes Dev.

[3] Hock MB, Kralli A (2009). Transcriptional control of mitochondrial biogenesis and function. Annu Rev Physiol.

[4] Spiegelman BM (2007). Transcriptional control of mitochondrial energy metabolism through the PGC1 coactivators. Novartis Found Symp.

[5] Calzone FJ, Hoog C, Teplow DB, Cutting AE, Zeller RW, Britten RJ, Davidson EH (1991). Gene regulatory factors of the sea urchin embryo. I. Purification by affinity chromatography and cloning of P3A2, a novel DNA-binding protein. Development.

[6] DeSimone SM, White K (1993). The Drosophila erect wing gene, which is important for both neuronal and muscle development, encodes a protein which is similar to the sea urchin P3A2 DNA binding protein. Mol Cell Biol.

[7] Becker TS, Burgess SM, Amsterdam AH, Allende ML (1998). Hopkins N: not really finished is crucial for development of the zebrafish outer retina and encodes a transcription factor highly homologous to human nuclear respiratory factor-1 and avian initiation binding repressor. Development.

[8] Schaefer L, Engman H, Miller JB (2000). Coding sequence, chromosomal localization, and expression pattern of Nrf1: the mouse homolog of Drosophila erect wing. Mamm Genome.

[9] Evans MJ, Scarpulla RC (1990). NRF-1: a trans-activator of nuclear-encoded respiratory genes in animal cells. Genes Dev.

[10] Scarpulla RC (1997). Nuclear control of respiratory chain expression in mammalian cells. J Bioenerg Biomembr.

[11] Gleyzer N, Vercauteren K, Scarpulla RC (2005). Control of mitochondrial transcription specificity factors (TFB1M and TFB2M) by nuclear respiratory factors (NRF-1 and NRF-2) and PGC-1 family coactivators. Mol Cell Biol.

[12] Dhar SS, Ongwijitwat S, Wong-Riley MT (2008). Nuclear respiratory factor 1 regulates all ten nuclear-encoded subunits of cytochrome c oxidase in neurons. J Biol Chem.

[13] Dhar SS, Liang HL, Wong-Riley MT (2009). Transcriptional coupling of synaptic transmission and energy metabolism: role of nuclear respiratory factor 1 in co-regulating neuronal nitric oxide synthase and cytochrome c oxidase genes in neurons. Biochim Biophys Acta.

[14] Dhar SS, Ongwijitwat S, Wong-Riley MT (2009). Chromosome conformation capture of all 13 genomic loci in the transcriptional regulation of the multisubunit bigenomic cytochrome C oxidase in neurons. J Biol Chem.

[15] Scarpulla RC (2012). Nucleus-encoded regulators of mitochondrial function: integration of respiratory chain expression, nutrient sensing and metabolic stress. Biochim Biophys Acta.

[16] Satoh J, Kawana N, Yamamoto Y (2013). Pathway analysis of ChIP-Seq-based NRF1 target genes suggests a logical hypothesis of their involvement in the pathogenesis of neurodegenerative diseases. Gene Regul Syst Bio.

[17] Hossain MB, Ji P, Anish R, Jacobson RH, Takada S (2009). Poly(ADP-ribose) polymerase 1 interacts with nuclear respiratory factor 1 (NRF-1) and plays a role in NRF-1 transcriptional regulation. J Biol Chem.

[18] Herzig RP, Andersson U, Scarpulla RC (2000). Dynein light chain interacts with NRF-1 and EWG, structurally and functionally related transcription factors from humans and drosophila. J Cell Sci.

[19] Hsiao HY, Jukam D, Johnston R, Desplan C (2013). The neuronal transcription factor erect wing regulates specification and maintenance of Drosophila R8 photoreceptor subtypes. Dev Biol.

[20] Huo L, Scarpulla RC (2001). Mitochondrial DNA instability and peri-implantation lethality associated with targeted disruption of nuclear respiratory factor 1 in mice. Mol Cell Biol.

[21] Ross JM. Visualization of mitochondrial respiratory function using cytochrome c oxidase/succinate dehydrogenase (COX/SDH) double-labeling histochemistry. J Vis Exp. 2011:e3266. 10.3791/3266 PMC3308593 22143245

[22] Wang SW, Mu X, Bowers WJ, Klein WH (2002). Retinal ganglion cell differentiation in cultured mouse retinal explants. Methods.

[23] Mao CA, Kiyama T, Pan P, Furuta Y, Hadjantonakis AK, Klein WH (2008). Eomesodermin, a target gene of Pou4f2, is required for retinal ganglion cell and optic nerve development in the mouse. Development.

[24] Schindelin J, Arganda-Carreras I, Frise E, Kaynig V, Longair M, Pietzsch T, Preibisch S, Rueden C, Saalfeld S, Schmid B (2012). Fiji: an open-source platform for biological-image analysis. Nat Methods.

[25] Chen H, Vermulst M, Wang YE, Chomyn A, Prolla TA, McCaffery JM, Chan DC (2010). Mitochondrial fusion is required for mtDNA stability in skeletal muscle and tolerance of mtDNA mutations. Cell.

[26] Li S, Chen D, Sauve Y, McCandless J, Chen YJ, Chen CK (2005). Rhodopsin-iCre transgenic mouse line for Cre-mediated rod-specific gene targeting. Genesis.

[27] Wong-Riley MT (2010). Energy metabolism of the visual system. Eye Brain.

[28] Furuta Y, Lagutin O, Hogan BL, Oliver GC (2000). Retina- and ventral forebrain-specific Cre recombinase activity in transgenic mice. Genesis.

[29] Wu F, Kaczynski TJ, Sethuramanujam S, Li R, Jain V, Slaughter M, Mu X (2015). Two transcription factors, Pou4f2 and Isl1, are sufficient to specify the retinal ganglion cell fate. Proc Natl Acad Sci U S A.

[30] Pan L, Deng M, Xie X, Gan L (2008). ISL1 and BRN3B co-regulate the differentiation of murine retinal ganglion cells. Development.

[31] Zhang XM, Yang XJ (2001). Regulation of retinal ganglion cell production by sonic hedgehog. Development.

[32] Dakubo GD, Wang YP, Mazerolle C, Campsall K, McMahon AP, Wallace VA (2003). Retinal ganglion cell-derived sonic hedgehog signaling is required for optic disc and stalk neuroepithelial cell development. Development.

[33] Edgar R, Domrachev M, Lash AE (2002). Gene expression omnibus: NCBI gene expression and hybridization array data repository. Nucleic Acids Res.

[34] Gao Z, Mao CA, Pan P, Mu X, Klein WH (2014). Transcriptome of Atoh7 retinal progenitor cells identifies new Atoh7-dependent regulatory genes for retinal ganglion cell formation. Dev Neurobiol.

[35] Mu X, Fu X, Beremand PD, Thomas TL, Klein WH (2008). Gene regulation logic in retinal ganglion cell development: Isl1 defines a critical branch distinct from but overlapping with Pou4f2. Proc Natl Acad Sci U S A.

[36] Mu X, Beremand PD, Zhao S, Pershad R, Sun H, Scarpa A, Liang S, Thomas TL, Klein WH (2004). Discrete gene sets depend on POU domain transcription factor Brn3b/Brn-3.2/POU4f2 for their expression in the mouse embryonic retina. Development.

[37] Pagliarini DJ, Calvo SE, Chang B, Sheth SA, Vafai SB, Ong SE, Walford GA, Sugiana C, Boneh A, Chen WK (2008). A mitochondrial protein compendium elucidates complex I disease biology. Cell.

[38] Calvo SE, Clauser KR, Mootha VK (2016). MitoCarta2.0: an updated inventory of mammalian mitochondrial proteins. Nucleic Acids Res.

[39] Wright AF, Chakarova CF, Abd El-Aziz MM, Bhattacharya SS (2010). Photoreceptor degeneration: genetic and mechanistic dissection of a complex trait. Nat Rev Genet.

[40] Ait-Ali N, Fridlich R, Millet-Puel G, Clerin E, Delalande F, Jaillard C, Blond F, Perrocheau L, Reichman S, Byrne LC (2015). Rod-derived cone viability factor promotes cone survival by stimulating aerobic glycolysis. Cell.

[41] Cronin T, Raffelsberger W, Lee-Rivera I, Jaillard C, Niepon ML, Kinzel B, Clerin E, Petrosian A, Picaud S, Poch O (2010). The disruption of the rod-derived cone viability gene leads to photoreceptor dysfunction and susceptibility to oxidative stress. Cell Death Differ.

[42] Virbasius JV, Scarpulla RC (1994). Activation of the human mitochondrial transcription factor a gene by nuclear respiratory factors: a potential regulatory link between nuclear and mitochondrial gene expression in organelle biogenesis. Proc Natl Acad Sci U S A.

[43] Koshiba T, Detmer SA, Kaiser JT, Chen H, McCaffery JM, Chan DC (2004). Structural basis of mitochondrial tethering by mitofusin complexes. Science.

[44] Meeusen S, McCaffery JM, Nunnari J (2004). Mitochondrial fusion intermediates revealed in vitro. Science.

[45] Meeusen S, DeVay R, Block J, Cassidy-Stone A, Wayson S, McCaffery JM, Nunnari J (2006). Mitochondrial inner-membrane fusion and crista maintenance requires the dynamin-related GTPase Mgm1. Cell.

[46] Taherzadeh-Fard E, Saft C, Akkad DA, Wieczorek S, Haghikia A, Chan A, Epplen JT, Arning L (2011). PGC-1alpha downstream transcription factors NRF-1 and TFAM are genetic modifiers of Huntington disease. Mol Neurodegener.

[47] Cam H, Balciunaite E, Blais A, Spektor A, Scarpulla RC, Young R, Kluger Y, Dynlacht BD (2004). A common set of gene regulatory networks links metabolism and growth inhibition. Mol Cell.

[48] Wang C, Li Z, Lu Y, Du R, Katiyar S, Yang J, Fu M, Leader JE, Quong A, Novikoff PM, Pestell RG (2006). Cyclin D1 repression of nuclear respiratory factor 1 integrates nuclear DNA synthesis and mitochondrial function. Proc Natl Acad Sci U S A.

[49] Poche RA, Zhang M, Rueda EM, Tong X, McElwee ML, Wong L, Hsu CW, Dejosez M, Burns AR, Fox DA (2016). RONIN is an essential transcriptional regulator of genes required for mitochondrial function in the developing retina. Cell Rep.

[50] Wang JL, Tong CW, Chang WT, Huang AM (2013). Novel genes FAM134C, C3orf10 and ENOX1 are regulated by NRF-1 and differentially regulate neurite outgrowth in neuroblastoma cells and hippocampal neurons. Gene.

[51] Agathocleous M, Love NK, Randlett O, Harris JJ, Liu J, Murray AJ, Harris WA (2012). Metabolic differentiation in the embryonic retina. Nat Cell Biol.

[52] Lunt SY, Vander Heiden MG (2011). Aerobic glycolysis: meeting the metabolic requirements of cell proliferation. Annu Rev Cell Dev Biol.

[53] Mathieu J, Ruohola-Baker H (2017). Metabolic remodeling during the loss and acquisition of pluripotency. Development.

[54] Domcke S, Bardet AF, Adrian Ginno P, Hartl D, Burger L, Schubeler D (2015). Competition between DNA methylation and transcription factors determines binding of NRF1. Nature.

[55] Westermann B (2010). Mitochondrial fusion and fission in cell life and death. Nat Rev Mol Cell Biol.

[56] Chen H, Chan DC (2005). Emerging functions of mammalian mitochondrial fusion and fission. Hum Mol Genet.

[57] Rai M, Katti P, Nongthomba U (2014). Drosophila Erect wing (Ewg) controls mitochondrial fusion during muscle growth and maintenance by regulation of the Opa1-like gene. J Cell Sci.

[58] Alexander C, Votruba M, Pesch UE, Thiselton DL, Mayer S, Moore A, Rodriguez M, Kellner U, Leo-Kottler B, Auburger G (2000). OPA1, encoding a dynamin-related GTPase, is mutated in autosomal dominant optic atrophy linked to chromosome 3q28. Nat Genet.

[59] Stone J, van Driel D, Valter K, Rees S, Provis J (2008). The locations of mitochondria in mammalian photoreceptors: relation to retinal vasculature. Brain Res.

[60] Bentmann A, Schmidt M, Reuss S, Wolfrum U, Hankeln T, Burmester T (2005). Divergent distribution in vascular and avascular mammalian retinae links neuroglobin to cellular respiration. J Biol Chem.

[61] Lovas JR, Wang X (2013). The meaning of mitochondrial movement to a neuron's life. Biochim Biophys Acta.

[62] Chang B, Hawes NL, Hurd RE, Davisson MT, Nusinowitz S, Heckenlively JR (2002). Retinal degeneration mutants in the mouse. Vis Res.

[63] Veleri S, Lazar CH, Chang B, Sieving PA, Banin E, Swaroop A (2015). Biology and therapy of inherited retinal degenerative disease: insights from mouse models. Dis Model Mech.

